# Emerging Mechanisms and Disease Implications of Ferroptosis: Potential Applications of Natural Products

**DOI:** 10.3389/fcell.2021.774957

**Published:** 2022-01-18

**Authors:** Chun Ge, Sujie Zhang, Huiwen Mu, Shaojun Zheng, Zhaoyi Tan, Xintong Huang, Chen Xu, Jianjun Zou, Yubing Zhu, Dong Feng, Jiye Aa

**Affiliations:** ^1^ Department of Pharmacy, Nanjing First Hospital, Nanjing Medical University, Nanjing, China; ^2^ Department of Clinical Pharmacy, School of Basic Medicine & Clinical Pharmacy, China Pharmaceutical University, Nanjing, China; ^3^ Key Laboratory of Drug Metabolism and Pharmacokinetics, State Key Laboratory of Natural Medicines, China Pharmaceutical University, Nanjing, China; ^4^ Department of Clinical Pharmacology, Nanjing First Hospital, Nanjing Medical University, Nanjing, China; ^5^ Nanjing Southern Pharmaceutical Technology Co., Ltd., Nanjing, China

**Keywords:** iron homeostasis, lipid peroxidation, redox signaling, cancer, neurodegenerative diseases, natural products

## Abstract

Ferroptosis, a newly discovered form of regulatory cell death (RCD), has been demonstrated to be distinct from other types of RCD, such as apoptosis, necroptosis, and autophagy. Ferroptosis is characterized by iron-dependent lipid peroxidation and oxidative perturbation, and is inhibited by iron chelators and lipophilic antioxidants. This process is regulated by specific pathways and is implicated in diverse biological contexts, mainly including iron homeostasis, lipid metabolism, and glutathione metabolism. A large body of evidence suggests that ferroptosis is interrelated with various physiological and pathological processes, including tumor progression (neuro)degenerative diseases, and hepatic and renal failure. There is an urgent need for the discovery of novel effective ferroptosis-modulating compounds, even though some experimental reagents and approved clinical drugs have been well documented to have anti- or pro-ferroptotic properties. This review outlines recent advances in molecular mechanisms of the ferroptotic death process and discusses its multiple roles in diverse pathophysiological contexts. Furthermore, we summarize chemical compounds and natural products, that act as inducers or inhibitors of ferroptosis in the prevention and treatment of various diseases. Herein, it is particularly highlighted that natural products show promising prospects in ferroptosis-associated (adjuvant) therapy with unique advantages of having multiple components, multiple biotargets and slight side effects.

## Introduction

Along with other biological processes, cell death is of great significance in various molecular physiological processes of mammalian development, homeostasis and disease (Thompson, 1995; Fuchs and Steller, 2011). Generally, cell death can be divided into “accidental cell death” (ACD) and “regulatory cell death” (RCD). ACD is the actual structural collapse of cells when exposed to extreme physicochemical or mechanical stimuli. However, RCD is initiated by a genetically encoded apparatus and can be altered by pharmacologic or genetic interventions. RCD plays an integral role throughout the normal development and lifespan of humans, eliminating irreversibly impaired, dysfunctional or infected cells to protect the tissue or organ system from environmental injury or infection. Based on different biochemical phenomena, RCD is identified as several subtypes, including apoptosis, necrosis, autophagy and so on (Galluzzi et al., 2015). In 2003, Dolma et al. discovered a non-apoptotic cell death process when exploring the selective toxicity of erastin (a small molecule) in cancer cells (Dolma et al., 2003). In 2007, Yagoda et al. determined mitochondrial voltage-dependent anion channels 2 and 3 (VDAC2/3) as specific targets of erastin and VDAC2/3 also induced a non-apoptotic cell death, which depends on the RAS-RAF-MEK pathway (Yagoda et al., 2007). In 2008, Yang and Stockwell et al. identified two compounds, RSL3 and RSL5, via a high-throughput method that selectively induced iron-dependent, non-apoptotic cell death in cancer cells with mutated oncogenic RAS subtype genes (Yang and Stockwell, 2008). In 2012, Dr. Brent R Stockwell et al. treated the human fibrosarcoma cell line HT-1080 with erastin and found that the subunit of the amino acid cysteine transporter on the cell surface was suppressed. Restriction of cystine import, glutathione (GSH) depletion and phospholipid peroxidase glutathione peroxidase 4 (GPX4) inactivation could result in cell death. Thus, the term “ferroptosis” was formally coined (Dixon et al., 2012). As numerous studies have reported, ferroptosis usually refers to a novel iron-dependent, lipid peroxidation-driven RCD, that is obviously distinct from apoptosis, necrosis and autophagy at the morphological, biochemical, and genetic levels (Xie et al., 2016a; Stockwell et al., 2017). For example, morphological features during ferroptosis include smaller mitochondria, increased membrane density, crest density and decrease/disappearance, plasma membrane blistering, and increased cytoplasmic and lipid peroxidation. Ferroptosis is not limited to neoplastic diseases, and has been mostly linked to other iron- or redox-associated pathophysiological conditions, such as neurodegeneration, ischemia/reperfusion (I/R) injury, diabetes, and immune dysfunction (Skouta et al., 2014). A large number of studies have demonstrated that ferroptosis plays an indispensable role in the occurrence, development and phenotypic shift of a variety of diseases, which may provide new diagnostic and therapeutic strategies to regulate cell survival and death. Hence, it is instructive to discover novel compounds and drugs regulating ferroptosis to expand the development of potential therapeutic approaches. In recent decades, many clinical drugs and experimental compounds have been exploited to modulate ferroptosis by preclinical and clinical studies so as to achieve therapeutic purposes. As studies have progressed, natural products have attracted sufficient attention. Natural products and their analogs, including traditional Chinese medicines (TCMs) are unneglectable and conventional sources for the discovery and development of modern drugs. Due to their natural origin and potential beneficial efficacy, natural products and herbal medicines have been used globally to promote human health concerns for thousands of years, especially for the therapy of many chronic diseases, such as cancer, cardiovascular and (neuro)degenerative diseases (Shu-Feng Zhou et al., 2007; Kibble et al., 2015). Emerging evidence has demonstrated solid ferroptotic bioactivities in natural compounds and herbal medicines. In this review, we discuss emerging molecular mechanisms and biological processes of ferroptosis and its role in various diseases. We summarize synthetic compounds and natural products with anti- or pro-ferroptotic properties, and the great potential of natural products for ferroptosis-associated (adjuvant) therapy is highlighted. Overall, the underlying mechanisms of ferroptosis and corresponding pharmacological agents can provide insights into the discovery and development of promising therapeutic strategies in terms of ferroptosis.

## Regulatory Networks and Signaling Pathways Associated With Ferroptosis

**FIGURE 1:**
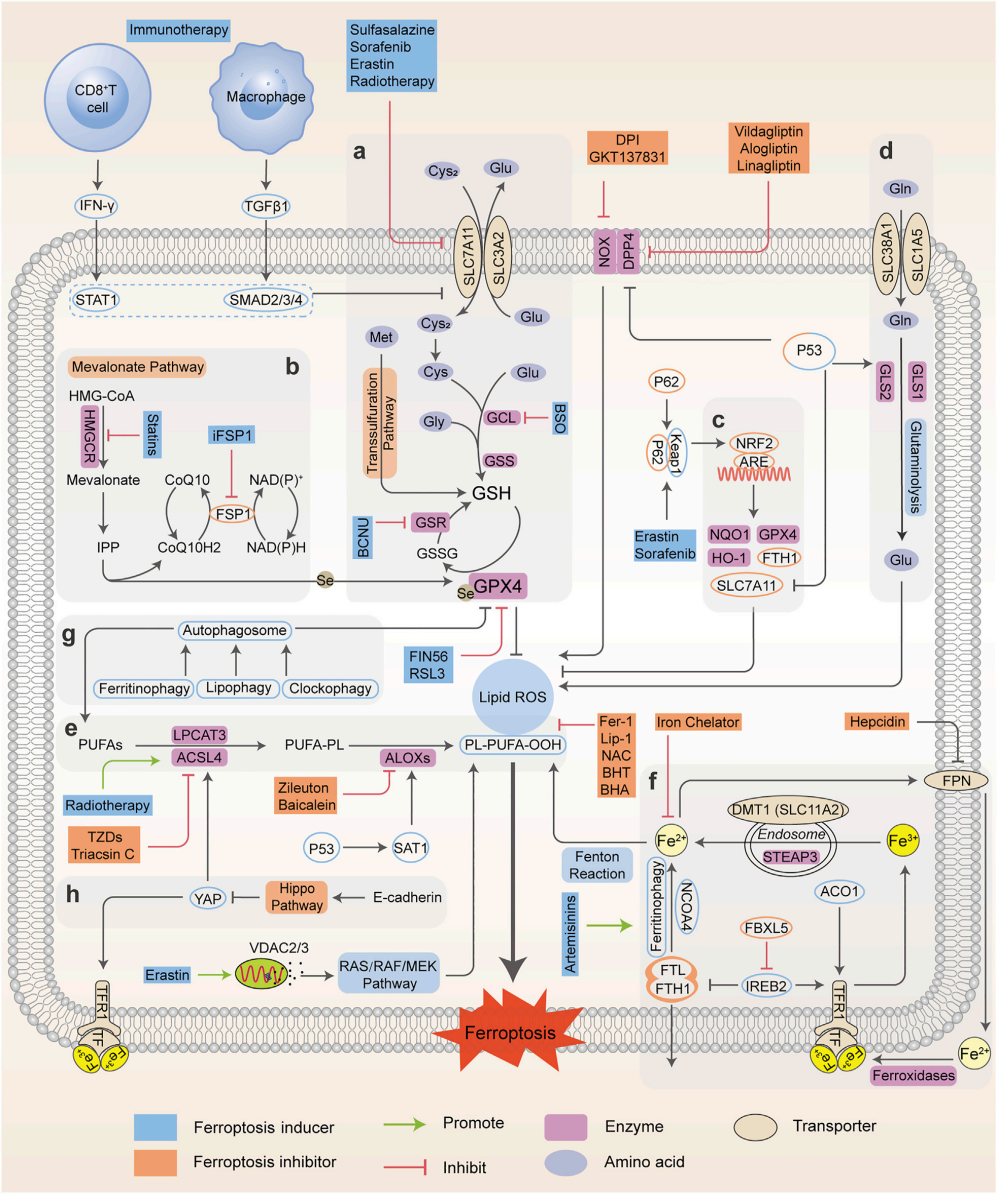
Regulatory networks and signaling pathways associated with ferroptosis. Ferroptosis is primarily activated by iron-dependent lipid peroxidation and redox perturbation, which mainly occurs through two major pathways, the extrinsic or transporter-dependent pathway, and the intrinsic or enzyme-regulated pathway. **(A)** The cyst(e)ine/GSH/GPX4 antioxidative axis. **(B)** The mevalonate pathway (IPP/FSP1/CoQ10 system). **(C)** NRF2-regulated ARE defence. **(D)** The glutaminolysis pathway. **(E)** The lipid peroxidation process; **(F)** The absorption, export, storage and utilization of iron. **(G)** The autophagy cascade. **(H)** The EMT-related pathway. Processes favoring or counteracting ferroptotic cell death are labeled with red and green arrows, respectively (Cys_2_, cystine; Cys, cysteine; Glu, glutamate; Gly, glycine Gln, glutamine; BCNU, 1,3-bis-(2-chloroethyl)-1-nitrosourea; CoQ10H2, ubiquinol; HMG-CoA, 3-hydroxy-3-methyl glutaryl coenzyme A; GSR, glutathione-disulfide reductase; BHA, butylated hydroxyanisole; BHT, butylated hydroxytoluene).

In recent years, the regulatory and metabolic mechanisms involved in ferroptosis have been emphatically elucidated and summarized by numerous original articles and reviews in detail. Hence, in this section, we briefly introduce the modulatory mechanisms and biological processes of ferroptosis, mainly including iron homeostasis, lipid metabolism, glutathione metabolism and other pathways, and illustrate them in Figure 1. Understanding the molecular mechanisms and signaling pathways of ferroptosis may open up new therapeutic opportunities to regulate cell fate in human diseases.

### Iron Homeostasis

Iron is of great physiological and biological functions such as the transport and storage of oxygen and energy metabolism. Normally, the intracellular iron maintains a balance within a narrow range. However, the disordered distribution and content of iron perturb physiological processes and biological survival. Excessive iron can provoke subsequent lipid peroxidation either by the iron-mediated Fenton reaction to produce reactive oxygen species (ROS, a group of molecules derived from molecular oxygen, mainly including hydrogen peroxide (H_2_O_2_), singlet oxygen (^1^O_2_), superoxide anion (O_2_^•–^) and hydroxyl radicals (^•^OH)), or the activation of enzymes containing iron (for example, lipoxygenases) (Xie et al., 2016a; Dixon and Stockwell, 2014). In general, the iron atom (Fe^3+^) binds to transferrin (TF) and binds with transferrin receptor (TFRC) through blood circulation to form the double iron-TF-TFRC complex, which is then endocytosed into the nucleus and reduced to ferrous (Fe^2+^) by endosomal six-transmembrane epithelial antigen of prostate 3 (STEAP3). Subsequently, Fe^2+^ is released from the nuclear endosome to the labile iron pool (LIP) in the cytoplasm via divalent metal transporter 1 (DMT1, SLC11A2). Excessive iron is stored in ferritin (the primary iron storage protein) which consists of ferritin light chain (FTL) and ferritin heavy chain 1 (FTH1). Finally, iron is exported from cells to blood through ferroportin (FPN, encoded by SLC40A1, the only known iron-efflux protein) in the cell membrane, and Fe^2+^ can be reoxidized by ferroxidases (such as ceruloplasmin or hephaestin) to Fe^3+^ (Torti and Torti, 2013). Alternatively, Fe^2+^ can be exported as ferritin through exosomes. Variable iron levels on account of interventions at multiple levels (such as iron absorption, storage, utilization, export and iron chelators) affect ferroptosis via an integrated signaling pathway (Figure 1). At the post-transcriptional level, iron regulatory protein 1 and 2 (IRP1; also known as ACO1, IRP2; also known as IREB2) can regulate the expression of DMT1, TFRC, ferritin and FPN (Torti and Torti, 2013) and thereby influence ferroptotic activity. Initiation of IREB2 promotes erastin-induced cytotoxicity, while inhibition of F-box and leucine-rich repeats protein 5 (FBXL5), an endogenous IREB2 antagonist, can sensitize cells to erastin (Reed and Pellecchia, 2012). Iron chelators, such as deferoxamine (DFO) (Dixon et al., 2012; Chen X. et al., 2020), dexrazoxane (DXZ) (Fang et al., 2019) and deferiprone (DFP) (Wu et al., 2020), can directly chelate iron and have been used in clinical treatment to reduce iron availability. As a result, these iron chelators are capable of relieving ferroptosis intrinsically or caused by proferroptotic reagents. Ferroptosis was originally identified as an autophagy-independent RCD (Xie et al., 2016a; Najafov et al., 2017). According to current studies, autophagy plays an important role in ferroptosis through the regulation of cellular iron levels. Autophagy recognizes and degrades ferritin into autophagosomes for lysosome-dependent degradation by using nuclear receptor coactivator 4 (NCOA4) as a selective cargo receptor. This process called “ferritinophagy”, which leads to increased iron accumulation and subsequent oxidative damage and ferroptosis. On the contrary, abrogating ferritinophagy by knockdown of autophagy-related genes or NCOA4 or inhibition of lysosomal function by specific inhibitors suppresses the availability of labile iron and decreases ferroptotic sensitivity (Mancias et al., 2014; Hou et al., 2016). In addition to NCOA4-mediated ferritinophagy, other forms of selective autophagy, including lipophagy, clockophagy, and chaperone-mediated autophagy, have been demonstrated to trigger ferroptosis in cells (Gao et al., 2016). Thus, intracellular iron homeostasis directly or indirectly targets ferroptosis, and the crosstalk of ferroptosis and autophagy provides new perspectives and contexts for the regulation of RCD.

### Lipid Peroxidation Metabolism

Lipid peroxidation has been regarded as the hallmark in the context of ferroptosis. As materials of lipid synthesis, polyunsaturated fatty acids (PUFAs), especially arachidonic acid (AA) and adrenic acid (AdA), are vulnerable to the peroxidation process and incorporated into membrane phospholipids (PLs), which destroy the lipid bilayer to trigger the ferroptotic process (Stockwell et al., 2017; Kagan et al., 2017; Conrad and Pratt, 2019). Acyl coenzyme A synthase long-chain member 4 (ACSL4) connects coenzyme A (CoA) to long-chain PUFAs, preferentially AA or AdA, to form arachidonic acid-CoA (AA-CoA) or adrenic acid-CoA (AdA-CoA), respectively. Then PUFA-CoAs are esterified by lysophosphatidylcholine acyltransferase 3 (LPCAT3) to produce PUFA-containing phospholipids (PUFA-PLs). Particularly, phosphatidylethanolamines (PEs) with both AA and AdA (AA/AdA-PEs) are the significant PUFA-PLs for ferroptosis, and are susceptible to free radical trigged oxidation catalyzed by lipoxygenases (ALOXs). ALOXs are nonheme iron-containing dioxygenases that catalyze the oxidation of PUFAs to produce fatty acid hydroperoxides, with a context-dependent role in mediating lipid peroxidation (Kagan et al., 2017; Doll et al., 2017) (Figure 1). Based on this, many reports have uncovered the implications of ACSL4, LPCAT3 and ALOXs during ferroptosis and exploited some candidate compounds with ferroptotic activities (Ou et al., 2016; Colakoglu et al., 2018). ACSL4 knockdown or inhibition can significantly lower the levels of AA-CoA and AdA-CoA to prevent the destruction of membrane structure, and confer ferroptosis resistance. Conversely, supplementation with AA or other PUFAs can block the original ACSL4-mediated reaction, which promotes the occurrence of ferroptosis or sensitizes cells to ferroptosis induced by erastin or RSL3 (Yang et al., 2016; Shintoku et al., 2017). However, the actual driver of lipid peroxidation and subsequent ferroptosis is still controversial, as it has been shown that ALOX15 is not essential for ferroptosis in a mouse model of GPX4-mediated acute renal failure (Friedmann Angeli et al., 2014).

As a master repressor of ferroptosis, selenoenzyme glutathione peroxidase 4, GPX4, consumes two molecules of reductive GSH after being combined with lipids to reduce toxic phospholipid hydroperoxide (LOOH) to nontoxic phospholipid lipid alcohol (LOH), which simultaneously produces oxidative glutathione (GSSG) as a byproduct, and ultimately inhibits phospholipid lipid peroxidation. Once they enter the membrane, PUFAs which are peroxidized and not cleared up by GPX4, eventually drive a large amount of lipid peroxidation and ferroptosis (Yang et al., 2016; Kagan et al., 2017). Further studies have shown that GPX4 inactivation is observed in the ferroptotic death process *in vitro* and *in vivo*, whereas deletion of GPX4 with pharmacological or genetic approaches can induce ferroptosis execution (Hangauer et al., 2017; Guo et al., 2018a). Besides western blot or RT-PCR evidence, many studies have confirmed the function and activity of GPX4 in the regulation of ferroptosis using cell or mouse models (e.g. GPX4 knockout or overexpression) (Alvarez et al., 2017; Hangauer et al., 2017; Viswanathan et al., 2017). For example, GSH-depleting reagents (erastin and buthionine sulfoximine (BSO)) inhibits GPX activity to induce ferroptosis and the total activity of GPXs in cells is examined using tert-butylhydroperoxide (tBuOOH) as a substrate. Furthermore, 7a-cholesterol-OOH is a specific substrate for GPX4; no other GPX enzyme can catalyze the reduction of 7a-cholesterol-OOH (1S, 3R)-RSL3, a ferroptosis inducer, has been demonstrated to inhibit enzyme activity of GPX4, indicated by unchanged level of 7a-cholesterol-OOH (Yang et al., 2014). Additionally, it is shown that FINO_2_ indirectly inhibits GPX4 enzymatic function while FIN56 directly depletes GPX4 enzymatic function using ^1^H-^15^N heteronuclear single quantum coherence (HSQC) NMR spectroscopy, ultimately causing widespread lipid peroxidation and ferroptosis (Gaschler et al., 2018a). It should be noted that the GPX4 defense system is also linked to other non-ferroptotic RCDs such as apoptosis and necroptosis, suggesting that lipid peroxidation lies at the crossroads of these pathways with diverse functions and different downstream effectors (Ran et al., 2007; Canli et al., 2016). Additionally, GPX4 is necessary for tumor recurrence, indicating that eradicating drug-resistant cancer cells by targeting GPX4 to induce cellular ferroptosis is a promising clinical strategy (Rennekamp, 2017).

### Glutathione Metabolism

The cystine-glutamate antiporter system x_c_^-^-mediated GSH synthesis, representative of the capacity of antioxidation, plays a significant role in the accumulation of lipid peroxides and the occurrence of ferroptosis. The cystine-glutamate antiporter, a transmembrane transport protein, composed of SLC7A11 (xCT) and SLC3A2 (4F2hc), takes up cystine from outside the cell and correspondingly outputs glutamate for exchange at a 1:1 ratio (Dixon et al., 2014). Usually, cysteine under reductive extracellular conditions is transported directly into cells through ASC (a neutral amino acid transport system), while cystine under oxidative extracellular conditions is reduced to cysteine after entering cells through system x_c_^-^. Subsequently, cysteine, glutamate, and glycine are catalyzed by γ-glutamylcysteine synthetase (GCLC) and glutathione synthase (GSS) respectively to form GSH, the major intracellular antioxidant (Doll and Conrad, 2017; Liang et al., 2019). The functional activity of GPX4 to reduce lipid hydroperoxides is dependent on GSH biosynthesis, because GSH acts as a cofactor for GPX4. GSH depletion leads to GPX4 inactivation, thereby triggering intracellular lipid peroxidation and ferroptosis. Therefore, system x_c_^-^ (SLC7A11) is a transportation hub that regulates intracellular redox balance and ferroptotic damage (Yang and Stockwell, 2016; Stockwell et al., 2017). The expression and activity of SLC7A11 is further positively modulated by nuclear factor erythroid 2-related factor 2 (NRF2), and negatively modulated by tumor suppressor genes, such as p53 and BAP1 (Carbone et al., 2013; Zhang Y. et al., 2018). This dual regulation constitutes a fine-tuning mechanism to delicately control and maintain GSH levels in ferroptosis. Consequently, ferroptosis is driven by GSH depletion either by restricting GSH biosynthesis (e.g., BSO inhibits GCLC) or by blocking cystine uptake from extracellular environment (e.g., erastin or sulfasalazine (SAS) inhibits system x_c_^-^). As an alternative way apart from system x_c_^-^, the transsulfuration pathway can supply cysteine as a compensation system under cystine deprivation, which is negatively regulated by cysteinyl-tRNA synthase (CARS). Loss of CARS has been proven to upregulate the transsulfuration pathway and inhibit erastin-induced ferroptosis (McBean, 2012; Hayano et al., 2016). Taken together, any link of GSH synthesis, representative of antioxidant capacity, can be a mediator of the ferroptotic cascade.

### Other Regulatory (co)factors and Signaling Pathways

#### p53

**FIGURE 2:**
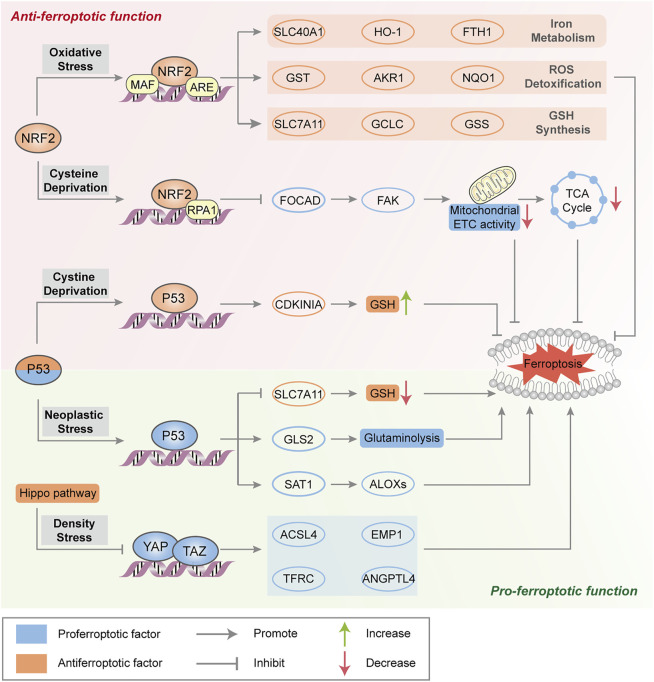
Major transcription factors in the regulation of ferroptosis. NRF2 transactivates a range of cytoprotective genes under endogenous oxidative stress to drive antiferroptotic function. Moreover, NRF2 represses FOCAD expression and FAK activity and further reduces sensitivity to cysteine deprivation-induced ferroptosis. Generally, p53 triggers ferroptosis to suppress tumorigenesis by transcriptional regulation of ferroptosis-related genes. In some circumstances, p53 suppresses metabolic stress-induced ferroptosis to preserve cell survival in certain cancer cells. The Hippo pathway negatively regulates a series of proferroptotic genes via YAP/TAZ transcription factors.

Emerging evidence suggests the two contradictory functions of the tumor suppressor p53 in the regulation of ferroptosis (Figure 2). On the one hand, p53 transcriptionally suppresses the expression of SLC7A11 in some types of cells (e.g., human breast or lung cancer cells) to trigger ferroptosis, which induces GSH depletion and relieves the specific inhibition of the ALOX12 enzyme to suppress tumor growth (Jiang et al., 2015; Chu et al., 2019). Moreover, p53-mediated transcriptional activation of spermidine/spermine N1-acetyltransferase 1 (SAT1) may indirectly increase ALOX15 expression, thus inducing lipid peroxidation and sensitizing cells to ferroptotic death (Ou et al., 2016). On the other hand, activation of p53 may antagonize ferroptosis in human colorectal cancer (CRC) cells and fibrosarcoma cells through various mechanisms, such as promoting the localization of dipeptidyl peptidase 4 (DPP4) to the nuclear enzyme-free pool in a transcription-independent manner, increasing the expression of CDKN1A (encoding p21) and favoring the preservation of GSH (Jennis et al., 2016; Xie et al., 2017). Considering that the transcription-dependent or independent function of p53 seems to be cell type-specific, it is necessary to evaluate the role of p53 in ferroptosis according to different circumstances.

#### NRF2

As a master protective regulator to maintain cellular redox homeostasis, NRF2 is unleashed from its ligand kelch-like ECH-associated protein 1 (Keap1) binding and translocates into the nucleus under oxidative stress. In the nucleus, NRF2 participates in the transcription of a series of antioxidant response element (ARE)-related genes that are implicated in iron metabolism (such as SLC40A1, heme oxygenase‐1 (HO-1) and FTH1), GSH synthesis (such as SLC7A11, GCLC and GSS) and ROS detoxification (such as aldosterone reductase family 1 (AKR1), glutathione thiotransferase (GST) and quinone oxidoreductase‐1 (NQO1)), resulting in the suppression of ROS- and/or iron-related ferroptosis (Sun et al., 2016a; Roh et al., 2017) (Figure 2). The p62-Keap1-NRF2 axis is engaged in oxidative stress-associated ferroptosis via the competitive binding of p62 to Keap1, which leads to the activation of NRF2 and downstream effectors. ARF is a crucial tumor suppressor, that activates the p53 pathway during carcinogenic stress (Sherr, 2006). Studies have shown that ARF blocks CBP (transcription coactivator)-dependent NRF2 acetylation and binds to its cognate transcriptional promoter, which inhibits NRF2-mediated transactivation (including SLC7A11) and leads to ferroptosis (Chen et al., 2017). In addition, NRF2 negatively regulates FOCAD gene expression in human non-small-cell lung cancer (NSCLC) cells, which is dependent on the NRF2-replication protein A1 (RPA1)-ARE complex. FOCAD is essential for focal adhesion kinase (FAK) activity, which further enhances the sensitivity of NSCLC cells to cysteine deprivation-induced ferroptosis via promoting the tricarboxylic acid (TCA) cycle and the activity of Complex I in the mitochondrial electron transport chain (ETC) (Liu P. et al., 2020). The role of NRF2 in ferroptosis sensitiviy and the therapeutic potential of NRF2 inhibitors need further investigation in ferroptosis-associated therapy.

#### Mitochondrial-Mediated Pathway

The contribution of mitochondria to the ferroptotic pathway is under intense debate. Some studies have shown that erastin alters the permeability of the outer mitochondrial membrane and directly binds to VDAC2/3 after cysteine deprivation (with erastin or cystine-free medium), leading to increased mitochondrial membrane potential (ΔΨ) and lipid peroxides (Bock and Tait, 2019; Gao et al., 2019). Other studies have shown that ROS production by the mitochondrial electron transport chain is not involved in the activation of ferroptosis (Gaschler et al., 2018b). Mitochondrial free iron accumulation can aggravate erastin-mediated ferroptosis and glutaminolysis is required for ferroptosis under cystine deprivation. Glutamine is degraded to glutamate catalyzed by glutaminase 1 and 2 (GLS1 and GLS2) in mitochondria and produces alpha-ketoglutaric acid (α-KG), which can effectively modulate ferroptotic death (Figure 1). Moreover, p53 can favor glutaminolysis by increasing GLS2 expression, which promotes ferroptosis through glutamate accumulation. Excessive extracellular glutamate decreases intracellular cysteine levels and eventually results in ferroptosis via inhibiting system x_c_^-^. Correspondingly, upregulation of GLS2 facilitates p53-dependent ferroptosis (Gao et al., 2015).

#### Mevalonate Pathway

The implication of the mevalonate pathway and ferroptosis has been well established, with two important products, isopentenyl pyrophosphate (IPP) and coenzyme Q10 (CoQ10) (Figure 1). Remarkably, IPP is essential for cholesterol biosynthesis, CoQ10 production and selenocysteine (Se)-tRNA function, which are responsible for the efficient translation of GPX4. Blocking 3-hydroxy-3-methylglutaryl coenzyme A reductase (HMGCR), the rate-limiting enzyme of the mevalonate pathway with statins can downregulate GPX4 activity and consequently induce the occurrence of ferroptosis (Shimada et al., 2016a; Viswanathan et al., 2017). Ferroptosis suppressor protein 1 (FSP1), previously known as ‘‘apoptosis-inducing factor mitochondria associated 2 (AIFM2)’’, promotes the production of CoQ10, an endogenous ferroptosis suppressor to restrain lipid peroxidation, which is independent of GPX4. Indeed, the FSP1-CoQ10-NAD(P)H signaling axis acts as an independent parallel system, that coordinates with GPX4 and GSH to combat oxidative stress and confer ferroptosis resistance (Bersuker et al., 2019; Doll et al., 2019).

#### Pentose Phosphate Pathway

Nicotinamide adenine dinucleotide phosphate (NADPH) serves as a principal reducing agent for biosynthesis across many biological processes, and is essential for maintaining intracellular GSH levels. Indeed, basal levels of NADPH are considered as a biomarker of ferroptosis susceptibility in several cancer cells, and NADPH depletion facilitates the ferroptotic cascade (Ding et al., 2020). The reduction in NADPH enhances erastin-, RSL3-and FIN56-induced ferroptosis (Shimada et al., 2016b). However, the pentose phosphate pathway (PPP) also produces NADPH as a substrate for NADPH oxidases (NOXs), which promote lipid peroxidation in subsequent ferroptosis. Direct inhibition of PPP or knockdown of related enzymes (glucose-6-phosphate dehydrogenase (G6PD) and phosphoglycerate dehydrogenase (PGD)) can enhance antioxidative activity to inhibit ferroptosis (Dixon et al., 2012; Hassannia et al., 2019). NADPH may play a dual role in ferroptotic regulation, which requires intensive explorations.

#### Epithelial-Mesenchymal Transition

Epithelial-mesenchymal transition (EMT) is identified that the epithelial phenotype with polarity and intercellular adhesion abilities progressively converts into the mesenchymal phenotype with migratory and invasive properties. EMT is an important cellular program during tumor migration, invasion and metastasis, contributing to drug resistance (Yang J. et al., 2020). EMT-related tumor metastasis and treatment resistance are provoked by transcription factors, such as SNAI1, TWIST1 and ZEB1, which are all potential targets in cancer therapy (van Staalduinen et al., 2018; Wu et al., 2019). Additionally, EMT has been demonstrated to be important for ferroptosis promotion. A highly mesenchymal-like cell state in human cancer cell lines and organoids confers a selective vulnerability to ferroptosis. Ferroptosis inhibitors, including RSL3, and statins have been reported to target the mesenchymal state for cancer therapy (Viswanathan et al., 2017). A CD44-dependent increase in iron endocytosis promotes the activity of iron-dependent demethylases, which increase the expression of genes related to EMT signaling, thereby sensitizing breast cancer cells to ferroptosis (Müller et al., 2020). Collectively, ferroptosis-based treatment may be effective in certain types of cancer, requiring careful consideration of cell type- and differentiation status-related pathways that may determine ferroptosis sensitivity and resistance.

#### Hippo Pathway

The Hippo pathway is usually believed to control cell number, organ size, tissue development and tumor growth, which negatively regulates the activity of several transcription factors, including transcription coactivators (e.g., yes-related protein 1 (YAP1) and WW domain containing transcription regulator 1 (WWTR1, also known as TAZ). As mentioned above, increased cell adhesion confers resistance to ferroptosis. In epithelial cells, neighboring cells interact through E-cadherin (ECAD) to activate NF2 (still identified as merlin) and the Hippo signaling pathway (Merlin-Hippo signaling) to promote ECAD-dependent cell adhesion, leading to ACSL4 transcriptional downregulation and ferroptosis resistance (Wu et al., 2019). In contrast, the activation of transcription factors involved in the Hippo pathway promotes ferroptosis in human renal cell carcinoma or ovarian cancer cells by regulating the expression of ferroptosis modulators (e.g., YAP-mediated ACSL4 and TFRC expression, TAZ-mediated epithelial membrane protein 1 (EMP1) and angiopoietin-like 4 (ANGPTL4) expression) to increase iron accumulation and lipid peroxidation (Yang et al., 2019; Yang Y. H. et al., 2020) (Figure 2).

## Ferroptosis and Its Implication in Diseases

**FIGURE 3:**
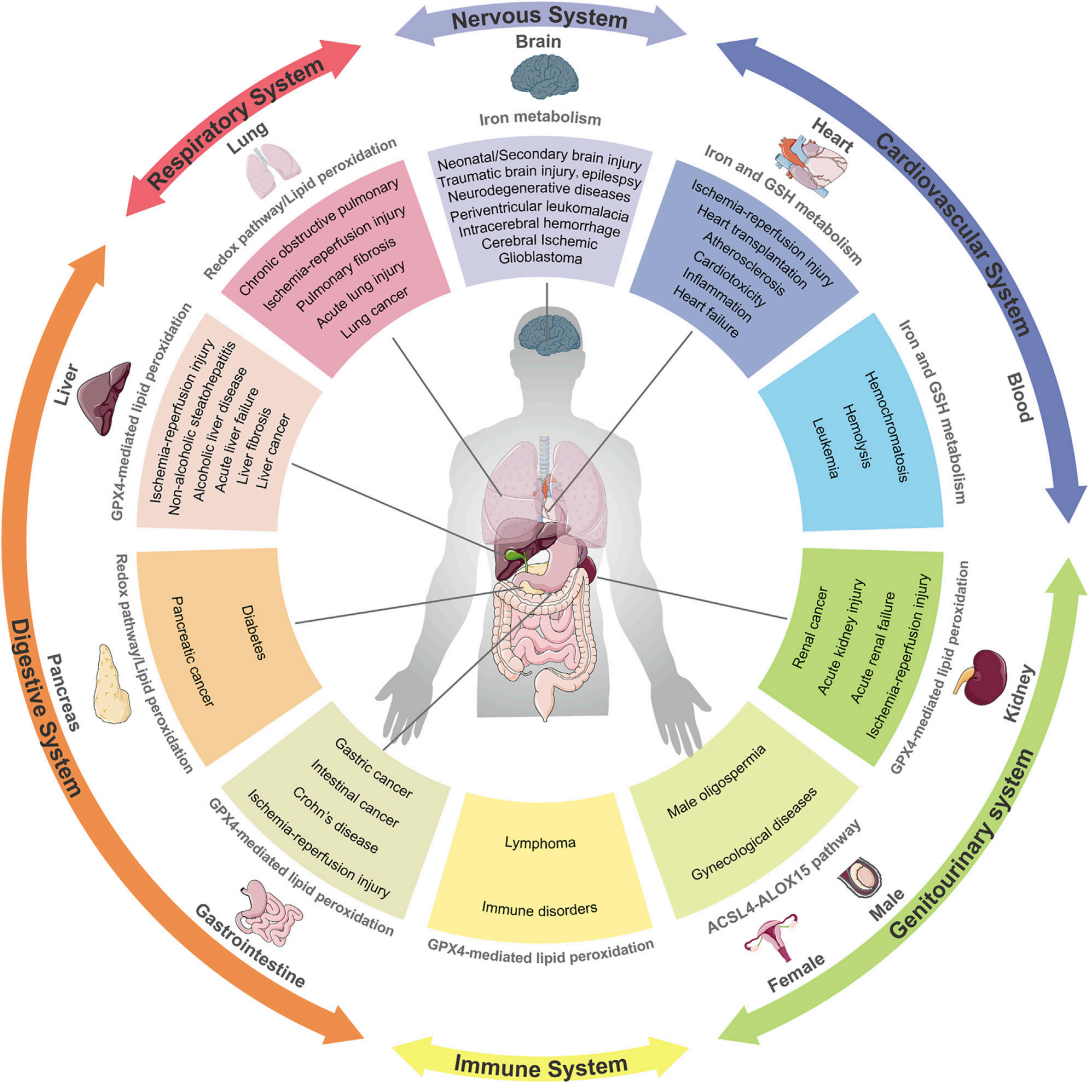
Ferroptosis and its implication in different pathophysiological contexts. Ferroptosis has been linked to pathological processes in diverse human body systems, including the nervous system, digestive system, respiratory system, circulatory system and urinary system. Abnormal pathways that contribute to diseases are recapitulatively presented.

The link between ferroptosis and human diseases has been mainly verified by either the impact of physiopathology on ferroptosis or the impact of ferroptosis on physiopathology. Indicators or hallmarks of ferroptosis (e.g., lipid peroxidation) in some pathological models are obviously altered consistently with the ferroptosis pathway. Alternatively, the pathological state varies with the induction or inhibition of ferroptosis. Either way, molecular mechanisms and signaling pathways involved in ferroptosis help to expand understanding of human health and diseases. Ferroptosis has been interrelated with multiple pathological and physiological environments, with a context-dependent role (Figure 3). On the one hand, ferroptosis, like other forms of RCD, plays a beneficial role in the maintenance of normal physiological functions in the body through the removal of excessively damaged or disordered cells during the response to environmental injury/infection. On the other hand, the death of useful or functional cells aggravates the initiation and progression of diseases, consequently leading to certain pathological states, such as cardiovascular disease and neurodegeneration. Hence, pathologies in diverse tissues or organ systems interrelated with ferroptosis and relevant research models are summarized in Table 1.

**TABLE 1:** The relevance of ferroptosis in diseases.

Diseases	Disease subtype	Test models	Impact of ferroptosis	Related effects and important findings in diseases	Refs
*Cancer*	Head and neck cancer (HNC)	A dozen of HNC cells; HN3R, HN9, HN9R HN10 xenograft mice Normal oral keratinocytes or fibroblasts obtained from patients	Ferroptosis of cancer cells inhibiting diseases	GPX4 inhibitors, (1S, 3R)-RSL 3 and ML-162 induce ferroptosis; Accumulated mitochondrial iron and lipid ROS promote ferroptosis	Roh et al. (2017), Kim et al. (2018), Shin et al. (2018)
	Breast *Cancer*	MDA-MB-231, T47D, HCC-1806, BT549, MCF-7(X) cells; TUBO, 4T1 xenograft mice; Patients’ samples		TRFC is a candidate marker of a subgroup of ER+/luminal-like breast cancer with poor outcome and tamoxifen resistance; GPX4-ACSL4 DKO cells show marked resistance to ferroptosis; Siramesine and lapatinib combination increase intracellular iron and ROS levels, and initially induce ferroptosis	Tonik et al. (1986), Habashy et al. (2010), Lanzardo et al. (2016), Ma et al. (2017), Yu et al. (2019)
	Hepatocellular carcinoma (HCC)	A dozen of HCC cells; THLE-3, HL-7702 primary human hepatocytes (PHH); Hepa1-6, Bel-7402 xenograft mice; DEN/CCl_4_-liver cancer model mice; Patients’ samples		The p62-Keap1-NRF2 pathway prevents ferroptosis and reduced GSH promote ferroptosis in liver cancer cells; Metallothionein-1 (MT-1), which inhibits lipid peroxidation, are associated with drug resistance and reduced overall survival; Ferroptosis inhibits liver tumorgenesis and is suppressed in liver cancer; XCT expression is higher, inversely related to the patient’s overall survival rate and disease-free survival rate	Kinoshita et al. (2013), Sun et al. (2016a), Sun et al. (2016b), Houessinon et al. (2016), Zhang X. et al. (2019), Bai et al. (2019)
	Lung cancer	A dozen of lung cancer cells; Mouse metastatic lung tumors		Lung adenocarcinomas select for expression of a pathway that confers resistance to high oxygen tension and protects cells from ferroptosis; Erastin upregulates p53 and inhibits SLC7A11, which induce ROS accumulation and ferroptosis	Wang S. J.et al. (2016), Alvarez et al. (2017)
	Gastric cancer (GC)	AGS, SGC7901, MGC803, MKN45 cells; BGC823 cells and xenograft mice; Patients’ samples		Cysteine dioxygenase 1 (CDO1) uptakes cysteine competitively, thereby restricting GSH synthesis and promoting ferroptosis; Suppression of CDO1 restores GSH level, prevents ROS production, upregulates GPX4 expression, and ultimately blocks lipid peroxidation and ferroptosis	Hao et al. (2017)
	Colorectal cancer (CRC)	TP53^+/+^ and TP53^−/−^ HCT116 cells and mice		Loss of p53 restricts the nuclear accumulation of DPP4 and thus facilitates plasma membrane-associated DPP4-dependent lipid peroxidation, which eventually leads to ferroptosis	Xie et al. (2017)
	Pancreatic cancer	MIAPaCa-2, CFPAC-1, BxPC-3, (resistant) PANC-1 cells		Ferroptosis inducer increases ROS production and activates ferroptosis; STAT3 is a positive regulator of ferroptosis and STAT3 silencing blocks erastin-induced ferroptosis	Kasukabe et al. (2016), Gao et al. (2018), Yamaguchi et al. (2018)
	Ovarian cancer	A dozen of ovarian cancer cells; HEY1 and HEY2 spheroids; ID8 cells and xenograft mice; Ovarian cancer cells isolated from patients		IFNγ cooperated with cyst(e)inase to increase lipid peroxidation and induce ferroptosis	Greenshields et al. (2017), Wang W. et al. (2019)
	Melanoma	SK-MEL-28 cells; A375, G-361, B16 cells and xenograft mice; Human melanoma cell lines established from patient biopsies		Inhibition of mitochondrial complex I triggers ROS production, lipid peroxidation and ferroptosis; Melanoma dedifferentiation increases sensitivity to ferroptosis; Depletion of cyst(e)ine and inhibition of system x_c_^−^ promote lipid peroxidation and ferroptosis; Expression of system xc^−^ is negatively associated with CD8^+^ T cell signature, IFNγ expression and patient outcome	Basit et al. (2017), Luo et al. (2018), Tsoi et al. (2018), Wang W. et al. (2019)
	Glioblastoma	F98, U87 cells; Glioblastoma patients		NRF2 level is inversely related to clinical outcome and overall survival; Fostered NRF2 expression and conversely Keap1 inhibition promote resistance to ferroptosis	Fan et al. (2017)
	Leukemia	Dozens of leukemia cells; Patient-derived xenografts (PDXs) of leukemia cells		High level of ACSL4 mRNA is expressed and is sensitive to ferroptosis; Low expression of FPN results in the susceptibility via increased iron levels; ROS produced by free ferrous iron leads to increased oxidative stress and ferroptosis	Yuan et al. (2016), Probst et al. (2017), Trujillo-Alonso et al. (2019)
	DLBCL; Renal cell carcinoma (RCC)	Dozens of DLBCL and RCC cells		DLBCL and RCC are particularly susceptible to GPX4-regulated ferroptosis; GPX4 is an essential mediator of ferroptotic cell death	Yang et al. (2014)
	Adrenocortical carcinoma (ACC)	NCI-H295R, HEK cells		Elevated expression of GPX4 and higher sensitivity to ferroptosis are found in ACCs	Belavgeni et al. (2019)
Neuro-degenerative diseases	Alzheimer’s disease (AD)	AD Patients; Brain tissues from GPX4BIKO mice; Tauopathy model mice	Ferroptosis of useful or functional cells inducing diseases	Iron-induced lipid peroxidation is abnormally elevated in the brain; Cerebrospinal fluid ferritin level is negatively correlated with cognitive ability; Ferroptosis inhibitors prevent neuronal damage	Ayton et al. (2015), Hambright et al. (2017), Zhang Y.-H. et al. (2018)
	Parkinson’s disease (PD)	PD Patients; LUHMES cells; Human brain tissues; MPTP-induced PD model mice		Iron concentration in the SN is related to the degree of disease progression and DFP improves related symptoms; Levels of MDA and lipid hydroperoxide are increased in the SN.	Devos et al. (2014), Pyatigorskaya et al. (2015), Do Van et al. (2016)
	Huntington’s disease (HD)	R6/2 HD mice; HD Patients		Plasma MDA, 4-hydroxynonenal (4-HNE) and lipid peroxidation are increased; IRPs 1/2, TFRC and GPX are decreased and FPN is increased	Klepac et al. (2007), Lee et al. (2011), Chen et al. (2013)
	Periventricular leukomalacia (PVL)	Oligodendrocytes		Fer-1 increases the number of healthy spinous neurons and inhibits oxidized lipid damage and ferroptosis	Skouta et al. (2014)
Brain diseases	Neonatal brain injury	Organotypic hippocampal slice cultures (OHSCs); Neonatal hypoxia-ischemia rats		Free iron is accumulated, TFRC expression is increased and ferritin expression is reduced	Lu et al. (2015)
	Traumatic Brain Injury (TBI)	TBI model HT22 cells; TBI model mice		AA/AdA-PE are increased; ALOX15, ACSL4 and GSH are exhausted; Ferroptosis inducers and mechanical stretch injury cause cell death	Kenny et al. (2019)
	Secondary brain injury (SBI)	Mouse brain astrocytes; ICH rats		GPX4 is downregulated in brain after ICH; GPX4 contributes to SBI following ICH by mediating ferroptosis; Induction of NRF2 expression serves as an adaptive self-defense mechanism	Cui et al. (2016), Zhang Z. et al. (2018)
	Intracerebral hemorrhage (ICH)	ICH mice; OHSCs; Human induced pluripotent stem cell (iPSC)-derived neurons		Fer-1 reduces iron accumulation, prostaglandin-endoperoxide synthase 2 (PTGS2) expression, lipid ROS and protects hemorrhagic brain from neuronal death	Li et al. (2017)
	Cerebral ischemia	MCAO mice and rats; Transient forebrain ischemia (TRI) rats		Ferritin, TFRC and iron accumulation are increased, and infarct focus is strengthened; The leaking blood-brain barrier (BBB) increases the iron level; Targeting iron-mediated oxidative stress holds extended therapeutic time window against an ischemic event	Park et al. (2011), Tuo et al. (2017)
Heart diseases	Ischemia-reperfusion (I/R)	Isolated hearts of mice; Cardiomyocytes		GSH level is significantly reduced and ROS level is increased; Inhibition of glutamate breakdown reduces I/R-induced heart damage; DFO improves function and reduces in myocardial infarcts size	Gao et al. (2015), Baba et al. (2018), Fang et al. (2019)
	Heart failure	Isolated adult cardiomyocytes; FPN knockout mice; Mice with cardiomyocyte-specific deletion of FTH1, hepcidin, or knock-in of hepcidin-resistant FPN		DXZ relieves myocardial toxicity; FTH1 deficiency leads to a decrease in cardiac iron level and an increase in oxidative stress; FPN knockout causes iron deposits in the myocardium and impairs cardiac function	Lakhal-Littleton et al. (2015), Lakhal-Littleton et al. (2016), Fang et al. (2020)
	Inflammation	Several immune deficient mice; Heart transplantation mice		Ferroptosis orchestrates neutrophil recruitment to injured myocardium by promoting adhesion of neutrophils to coronary vascular endothelial cells through TLR4/TRIF signaling pathway, which inhibited by Fer-1	Li W. et al. (2019)
	Atherosclerosis	Overexpressing GPX4 and control Apolipoprotein E (ApoE)^−/−^ mice		Iron accumulation causes ROS accumulation and death in macrophages; Increased antioxidant capacity can reduce the ferroptosis of macrophages; GPX4 overexpression inhibits plaque formation by inhibiting oxidized lipid modification and reduces mid-advanced aortic sinus lesions	Guo et al. (2008)
Blood diseases	Hemolysis	J774 cells; RBC transfusion and clearance model mice		Increased red blood cells (RBCs)through phagocytosis lead to iron degeneration, ROS accumulation and lipid peroxidation in splenic red plasma macrophage (RPMs), which can be ameliorated by Fer-1; Ferroptosis may be clinically relevant to transfusion-related immunomodulation and impaired host immunity	Youssef et al. (2018)
	Hereditary hemo-chromatosis (HH)	Primary hepatocytes; Bone marrow-derived macrophage (BMDMs); SLC7A11^−/−^ mice; HH model mice		Iron overload is sufficient to trigger ferroptosis both *in vitro* and *in vivo*; SLC7A11 confers protection against ferroptosis during iron overload; SLC7A11 depletion facilitates ferroptosis onset specifically under high-iron conditions	Wang et al. (2017a)
Lung diseases	Chronic obstructive pulmonary (COPD)	Human bronchial epithelial cells (HBECs); BEAS-2B, A549 cells; GPX4 deficient or transgenic mice		Cigarette triggers NCOA4-mediated ferritinophagy; Iron accumulation and lipid peroxidation are increased, which can be reversed by GPX4 knockout, DFO and Fer-1	Yoshida et al. (2019)
	Pulmonary I/R	Pulmonary I/R model mice; Hypoxia-reoxygenation model A549 cells		ACSL4 expression is enhanced and GPX4 expression is reduced; Ferroptotic features emerge after lung I/R injury, which is prevented by liproxstatin-1 (Lip-1)	Xu et al. (2020)
Liver diseases	Acute liver failure	ACSL4 KO mice; acetaminophen (APAP)-induced acute liver failure mice		APAP administration induces hepato-toxicity, lipid peroxidation, PTGS2 upregulation and GSH depletion, which are markedly suppressed by Fer-1and DFO.	Yamada et al. (2020b), Yamada et al. (2020c)
	Non-alcoholic steatohepatitis (NASH)	Several NASH model mice; CCl_4_ induced liver injury mice		Enhanced AA metabolism, iron-mediated lipid ROS accumulation, mitochondrial morphological changes are alleviated by ferroptosis inhibitors	Tsurusaki et al. (2019), Li X. et al. (2020)
	Alcoholic liver disease (ALD)	ALD patients		Serum hepcidin is decreased; Iron, ferritin and FPN are upregulated	Dostalikova-Cimburova et al. (2014)
	Hepatic I/R; Living donor liver transplantation (LDLT)	Hepatic I/R model mice; Hepatic I/R injury in pediatric LDLT		A high serum ferritin level, a marker of iron overload, is an independent risk factor for liver damage after LT; Liver damage, lipid peroxidation, and upregulation of PTGS2 are induced by I/R	Yamada et al. (2020a)
Pancreas diseases	Diabetes mellitus and its complications	NRK-52E cells; Type 2 diabetes (T2DM) mice; Diabetic nephropathy mice		Depleted GSH and downregulated GPX4 induce oxidative stress in pancreatic tissue of T2DM molding; ACSL4 is increased and GPX4 is decreased in DN mice	Li D. et al. (2020), Wang et al. (2020)
Gastrointestinal diseases	Intestinal I/R	Caco-2 cells; Intestinal I/R model mice		ACSL4 and cyclooxygenase 2 (COX2) are increased while GPX4 and FTH1 are reduced in I/R-induced intestinal injury	Li Y. et al. (2019)
	Crohn’s disease (CD)	GPX4 deficient intestinal epithelial cells (IECs); GPX4^+/−IEC^ mice; CD patients		IECs in CD exhibit impaired GPX4 activity and signs of lipid peroxidation	Mayr et al. (2020)
Kidney diseases	Acute kidney injury; Acute renal failure (ARF)	Human renal proximal tubule epithelial cells (HRPTEpiCs); GPX4^−/−^ Pfa1 cells; GPX4^−/−^ mice		Mitochondrial lipid phosphatidylcholine (PC), PE and cardiolipin are heavily oxidized; Ferroptosis inhibitor, SRS16-86 strongly protects kidneys	Friedmann Angeli et al. (2014), Skouta et al. (2014)
Immune diseases	Immune disorders	GPX4-deficient T cells; T cell-specific GPX4 deficient mice; Peripheral blood mononuclear cell (PBMCs)		GPX4 deficiency causes T cells to fail to protect against viruses and infections, which can be rescued by vitamin E; Rapid accumulation of membrane lipid peroxides induces ferroptosis; Erastin-induced lipid peroxidation promotes PBMCs proliferation and differentiation into B cells and natural killer cells	Matsushita et al. (2015), Wang D. et al. (2018)
Other diseases	Age-related macular degeneration (AMD)	ARPE-19 cells		Oxidative stress-mediated senescence upon GSH depletion and subsequent death of photoreceptors are observed in AMD.	Sun et al. (2018)

### Cancer

*Cancer* is a multifactorial and complicated disease that contributes to a major health burden worldwide, despite substantial efforts and considerable improvement. In general, there are two key challenges in cancer treatment. One is how to kill cancer cells effectively without affecting healthy cells. Second, cancer cells in advanced tumors often exhibit multiple genetic variations with high oxidative stress and enhanced antioxidant capacity, which confer drug resistance (Trachootham et al., 2009). Ferroptosis, as a novel form of cell death, offers unique possibilities efficiently against a variety of cancers. A higher iron requirement than their non-malignant counterparts has been observed to support sustained proliferation and immortalized replication in certain cancer cells, along with FPN downregulation and TFRC upregulation. The strong dependency on iron makes cancer cells more susceptible to iron overload and ROS-mediated lipid peroxidation than noncancerous cells. Epidemiological investigations have also revealed that high dietary iron intake increases the risk of several cancer types (such as hepatocellular carcinoma (HCC) and breast cancer) (Fonseca-Nunes et al., 2014). Ferroptosis inducers have been proven effective in suppressing the occurrence and development of cancers derived from iron-rich tissues, such as HCC, breast cancer and pancreatic ductal adenocarcinoma (PDAC) (Gao et al., 2015; Friedmann Angeli et al., 2019). According to a sensitivity profiling in 177 cancer cell lines, renal cell carcinomas (RCCs) and diffuse large B cell lymphomas (DLBCLs) are particularly susceptible to GPX4-regulated ferroptotic cell death (Yang et al., 2014). From another perspective, it is well acknowledged that the activation of the apoptosis cascade is the most prevalent way to eliminate cancer cells. Unfortunately, the apoptotic pathway of most mutant cancer cells is frequently blocked, which confers robust drug resistance (Amable, 2016). *Cancer* cells resistant to some forms of death may still be vulnerable to the induction of other forms of cell death, suggesting bypass of apoptotic resistance is a considerable approach to combating resistant cancers. A large amount of evidence has confirmed that ferroptosis induction plays a predominant role against certain chemoresistant cancer cells that are in a high mesenchymal state or escaping drug treatment (Holohan et al., 2013). Recent research has showed that cisplatin can induce both ferroptosis and apoptosis in the NSCLC A549 cells and colon cancer HCT116 cells, while drug resistance is observed in cisplatin-induced apoptosis, rather than ferroptosis (Guo et al., 2018a). Additionally, some drug-resistant cancer cells with activated EMT signaling (upregulation of mesenchymal markers and downregulation of epithelial markers) become specifically sensitive to ferroptosis-based therapies (Viswanathan et al., 2017; Müller et al., 2020). Beyond acting as a monotherapy, ferroptosis induction also participates in combination with conventional therapies. For instance, erastin, a classical ferroptosis inducer, has been shown to enhance the chemotherapeutic efficacy of traditional anticancer drugs, such as temozolomide and cisplatin in specific cancer cell lines (Yamaguchi et al., 2013; Chen et al., 2015). Accordingly, deficiencies in a host of NRF2 targets, including GPX4, SLC7A11 and FTH1/FTL, are also capable of predisposing cells to succumb to proferroptotic agents in many cancer types (Hassannia et al., 2019).

Currently, discovered proferroptotic compounds mainly spotlight on key elements of redox homeostasis (e.g., system x_c_^-^ and GPX4) to disrupt the existing redox balance and trigger the ferroptotic cascade in cancers whose growth and survival are highly dependent on the uptake of amino acids. Lack of SLC7A11 and knockdown of GPX4 have been demonstrated to increase ROS-mediated lipid peroxidation and induce subsequent ferroptosis in even resistant cancer cells, indicating that deficiency in antiferroptotic function enhances cancer cell death (Yang et al., 2014; Roh et al., 2016). Furthermore, increased expression of SLC7A11, GPX4 or their upstream regulatory gene NRF2 not only promotes ferroptosis resistance, but is also correlated with a poorer prognosis and lower survival rate in many types of cancers, such as liver, lung and ovarian cancers (Fan et al., 2017; Daher et al., 2019; Lim et al., 2019). However, the role of ferroptosis in tumorigenesis and progression remains elusive, as ferroptosis appears to have a dual role. There has been evidence that deficiency in NRF2 favors early tumorigenesis in several cancers (Rojo de la Vega et al., 2018). Another critical mediator of the proferroptotic cascade, ACSL4, is downregulated in bladder, brain, breast, leukemia and lung cancer but upregulated in colorectal, head and neck, kidney, myeloma and liver cancer. Prognostic analysis shows that colorectal cancer patients with high ACSL4 expression have a low survival rate. In contrast, brain, breast and lung cancer patients with low ACSL4 expression have a poor survival, suggesting that ACSL4 plays different roles in different cancer types (Chen et al., 2016).

With a comprehensive understanding, ferroptosis has also been implicated in cancers undergoing immunotherapy and radiotherapy. Immunotherapy with immune checkpoint blockade, which activates an effective cytotoxic T cell-triggered antitumor immune-response, has a revolutionary impact on clinical cancer treatment. Mechanistically, interferon gamma (IFNγ) released from CD8^+^ T cells can impair cystine uptake by cancer cells upon downregulating the expression of SLC3A2 and SLC7A11 (two subunits of the cystine-glutamate antiporter system x_c_^-^) and consequently promote lipid peroxidation and ferroptosis in ovarian cancer, melanoma and fibrosarcoma (Tsoi et al., 2018; Wang W. et al., 2019; Lang et al., 2019). Radiotherapy acts as frontline therapy for approximately half of patients with many cancers. Recently, ferroptosis has also been proven to be interrelated with cancer radiotherapy, which induces ACSL4 expression and enhances oxidative damage in a variety of cancers, including NSCLC, melanoma and esophageal cancer (Lang et al., 2019; Lei et al., 2020). Indeed, immunotherapy and radiotherapy synergistically activate tumoral lipid oxidation and ferroptosis.

However, ferroptosis also triggers severe damage and toxicity in organisms, including bone marrow injury and cisplatin-induced acute kidney injury. How to modulate ferroptosis pathway to obtain the highest clinical benefit in clinical oncology is a critical question that deserves further investigation.

### Neurodegeneration and Brain Injury

A large body of evidence suggests that low levels of GSH/GSSG, elevated iron levels and lipid peroxidation are implicated in a variety of neurodegenerative diseases, along with abnormal levels of ferroptosis-associated modulators (Praticò and Sung, 2004; Belaidi and Bush, 2016). Alzheimer’s disease (AD) is the most common neurodegenerative disease and is characterized by synaptic loss and neuronal death. In AD patients, there is abnormally elevated iron in the brain tissue, and ferritin levels are negatively correlated with cognitive ability (Ayton et al., 2015). Excessive ROS generation triggers lipid peroxidation, which contributes to severe oxidative stress damage and is observed in GPX4-deficient AD mouse models, while ferroptosis inhibitors ameliorate neurodegeneration and behavioral dysfunction by blocking iron overload, lipid peroxidation and inflammation (Hambright et al., 2017; Zhang Y.-H. et al., 2018). Parkinson’s disease (PD) is characterized by progressive dopaminergic neuronal loss in the substantia nigra (SN) pars compacta. Epidemiological investigations have shown that high iron levels lead to a high risk of PD, associated with enhanced basal lipid peroxidation and elevated malondialdehyde (MDA) levels, a toxic byproduct of lipid peroxidation (Pyatigorskaya et al., 2015). Given the implication of iron in PD, preclinical experiments and clinical trials have revealed that the U.S. Food and Drug Administration (FDA)-approved iron chelators improve dopamine activity and prevent iron-mediated oxidative damage, by exerting a certain protective effect in PD patients (Devos et al., 2014; Chen X. et al., 2020). Several features of Huntington’s disease (HD) physiopathology are consistent with the ferroptosis pathway, including a lower GSH level, a higher lipid oxidation level and abnormal iron metabolism. Accordingly, administration of DFO significantly improves striatal pathology and motor phenotype in an R6/2 HD mouse model (Klepac et al., 2007; Lee et al., 2011; Chen et al., 2013). Additionally, ferrostatin-1 (Fer-1, classical ferroptosis inhibitor) and its analogs inhibit lipid peroxidation damage and increase the number of healthy spinous process neurons in the HD model. Apart from common neurodegeneration, other neurodegenerative disorders are also characterized by perturbations in iron homeostasis and accumulation, such as periventricular leukomalacia (PVL) (Skouta et al., 2014), amyotrophic lateral sclerosis (ALS) (Southon et al., 2020), multiple sclerosis (MS) (Jhelum et al., 2020), Friedreich’s ataxia (FRDA) (Cotticelli et al., 2019). Affected tissue regions share features of elevated iron, low GSH, decreased GPX4, as well as increased lipid peroxidation, consistent with the salient features of ferroptosis. Although there are few relevant studies, some ferroptosis-associated factors appear to be potential markers and therapeutic targets in these disorders (Hu et al., 2019; Seco-Cervera et al., 2020).

Besides leading to neurodegenerative diseases, the activation of the of ferroptotic cascade has also been determined in a wide variety of neuronal injuries. Neonatal brain injury, as well as traumatic brain injury (TBI), is usually accompanied by a series of biochemical processes, such as dysfunctional iron metabolism, decreased GPX activity, ROS accumulation and disordered ferroptosis-related mediators *in vitro* and *in vivo* (Lu et al., 2015; Kenny et al., 2019). Oxidative stress damage induced by specific GPX4 deletion in the brain is observed during the acute phase of intracerebral hemorrhage (ICH) and subsequent secondary brain injury (SBI) and finally leads to hemorrhagic stroke which can be relieved by an increasing GPX4 levels. The application of N-acetylcysteine (NAC, a ROS scavenger) or Fer-1 has been documented to prevent neuronal mortality after ICH and improve the prognosis of patients, by decreasing iron accumulation and lipid ROS (Li et al., 2017; Zhang Z. et al., 2018). Dysfunction of tau protein contributes to age-dependent, iron-mediated neurotoxicity in ischemic stroke tissue. Tau is capable of blocking ferroptosis by promoting iron export against middle cerebral artery occlusion (MCAO)-induced focal cerebral ischemia-reperfusion injury. Ferroptosis has been recognized as a main cause of neuronal death after ischemic stroke, and ferroptosis inhibitors significantly improve the prognosis of patients (Tuo et al., 2017). Actually, ferroptosis inhibitors have been successfully applied in animal models of I/R-associated tissue injuries including heart (Fang et al., 2019), lung (Xu et al., 2020), liver (Yamada et al., 2020a), intestine (Li Y. et al., 2019) and kidney (Friedmann Angeli et al., 2014).

Overall, these studies suggest that ferroptosis irreversibly drives neuronal loss and tissue damage linked with central and/or the peripheral neuronal pathologies. The effectiveness of ferroptosis inhibition as a therapeutic approach has been mostly validated by cellular and animal experiments. However, there seems to be a lack of strong evidence in clinical trials. Providing comprehensive treatments for neurological diseases remains a largely insurmountable challenge.

### Cardiovascular Diseases

As “iron-induced heart disease” was first reported, iron-induced ferroptosis has been gradually identified as playing a detrimental role in the occurrence and aggravation of cardiovascular conditions in both animal models and human patients (Sullivan, 1981). Abnormal myocardial iron accumulation plays a pathogenic role in the myocardium which leads to cardiotoxicity and cardiac dysfunction. Studies have shown that ferroptosis mediates myocardial damage caused by doxorubicin (DOX) or I/R mainly by HO-1 upregulation, and subsequently degrading heme to release free iron and generate mitochondrial membrane oxidized lipids. Iron deposition in the myocardium of FPN knockout mice which results in severe damage to cardiac function has been recognized as a risk factor for coronary artery diseases (Bagheri et al., 2013; Lakhal-Littleton et al., 2015). Iron chelators, such as DFO and DFP have been shown to counteract the pathogenic effects of iron overload, indicating a promising therapeutic strategy against ferroptosis-driven cardiac abnormalities (Gao et al., 2015; Fang et al., 2019). Nonetheless, cardiomyocyte iron deficiency can also lead to fatal contractile and metabolic dysregulation as a consequence (Lakhal-Littleton et al., 2016). This findings supports compelling evidence that the influence of ferroptosis on cardiac diseases depends on proper iron homeostasis, rather than absolutely low iron levels. Ferroptosis has also been involved in inflammatory responses after heart transplantation, which promotes the adhesion of neutrophils to coronary endothelial cells and myocardial I/R damage. Fer-1 can alleviate the loss of cardiomyocytes and limit the recruitment of neutrophils by the TLR4/TRIF signaling pathway (Li W. et al., 2019). As the master regulator of systemic iron homeostasis, hepcidin is derived primarily from the liver, and it inhibits the iron exporter FPN to decrease iron availability in blood (Lakhal-Littleton et al., 2016). Due to an inherited genetic disorder or receiving multiple blood transfusions, the depletion of hepcidin induces ferroptosis as a result of iron overload in the body. As an iron overload disease that blocks hepcidin biosynthesis, hemochromatosis can be ameliorated by either iron chelators or genetic regulation of ferroptosis, such as targeting SLC7A11 (Wang H. et al., 2017).

Although the link with cardiovascular diseases still requires further evaluation by clinical evidence, iron-induced ferroptosis has been well-established as a master trigger of several cardiovascular pathologies. Chelation therapy has been considered as an efficient preventative measure and treatment approach and the key is how to maintain relatively precise iron homeostasis.

In addition to common diseases mentioned above, more pathologies associated with ferroptosis are summarized in Table 1 and Figure 3.

## Pharmacological Approaches to Ferroptosis Modulation

A more profound understanding of the regulatory and metabolic mechanisms of ferroptosis in a variety of diseases provides attractive approaches to diagnosis and therapy. As mentioned above, the positive or negative role of ferroptosis depends on the context of diseases with different pathological and physiological processes. In cancer therapy, it aims to slow or suppress tumor growth by inducing ferroptosis in cancer cells without affecting non-malignant cells. Thus, the activation of ferroptosis appears to be a promising therapeutic strategy to block pro-carcinogenic alterations, especially those are resistant to other modes of cell death. In degenerative or traumatic diseases, such as neurodegenerative disorders and I/R injury, the therapeutic goal is to protect functional cells from ferroptosis-induced injury. As such, ferroptosis inhibitors are expected to reverse or mitigate oxidative damage and disease progression and even achieve curative effect. Besides individual effects, multiple ferroptosis regulators can also act synergistically with some conventional drugs. Many kinds of synthetic compounds and natural products have been identified as inducers and inhibitors of ferroptosis to overcome diseases by modulating ferroptotic or mixed death.

### Synthetic Compounds and Their Modulation on Ferroptosis

Currently, numerous chemical reagents with ferroptotic properties have been continuously exploited and demonstrated in preclinical and clinical experiments. Although the underlying mechanism needs more investigation, ferroptosis inducers are reckoned as a novel therapeutic strategy against various cancers, especially those resistant to conventional drugs. Indeed, ferroptosis was first discovered by studying reaction mechanisms of two small RAS-selective molecule compounds, erastin and RSL3, including their derivatives, that have been confirmed to suppress tumor growth in many types of cancers *in vitro* and *in vivo* (Dixon et al., 2012; Yang et al., 2014; Viswanathan et al., 2017; Zhang Y. et al., 2019). Originally, ferroptosis-inducing reagents were divided into two mechanistic classes: 1) inhibitors of cystine via system x_c_^-^ (e.g., erastin, sorafenib, SAS and glutamate), which subsequently drive GSH depletion, and 2) direct inhibitors of GPX4 (e.g., RSL3 (1S, 3R)-RSL3). More recently, the small molecule FIN56 has been reported to degrade GPX4 and to deplete CoQ10, an endogenous inhibitor of lipid peroxidation, through the mevalonate pathway. Moreover, FINO_2_ can either oxidize ferrous iron directly, or deactivate GPX4 indirectly (Shimada et al., 2016a; Gaschler et al., 2018a). On the contrary, ferroptosis also participates in other nonneoplastic lesions and a variety of compounds can pharmacologically inhibit ferroptosis, most of which are iron chelators or antioxidants. Iron chelators (e.g., DFO and DXZ) or regulators of iron metabolism suppress ferroptosis through lowering free iron levels (Fang et al., 2019; Chen X. et al., 2020). In addition, inhibitors of lipid peroxidation, including ACSL4 inhibitors (e.g., thiazolidinediones (TZDs)), lipophilic antioxidants (e.g., α-tocopherol), and agonists of the antioxidant system (e.g., ROS scavengers (NAC) and system xc^-^ inducers (β-mercaptoethanol)) inhibit ferroptosis via single or complex mechanisms (Dixon et al., 2012; Karuppagounder et al., 2018).

Fortunately, some of these pro- and anti-ferroptotic compounds are clinical drugs approved by the FDA, and are expected to be novel therapeutic applications based on ferroptotic mechanisms. Mechanistically, SAS, sorafenib and lanperisone have been broadly documented to inhibit cysteine-glutamate antiporter and cystine absorption, leading to the attenuation of GSH, and ultimately resulting in the death of certain types of cancer cells, including HCC, lymphoma and prostate cancer (Lachaier et al., 2014; Sun et al., 2016a; Sun et al., 2016b). Siramesine, lapatinib and salinomycin (ironomycin) can trigger ferroptosis by elevating iron levels in breast cancer cells and leukemia cells (Hamaï et al., 2017; Ma et al., 2017; Mai et al., 2017). Iron chelators are usually used as antidotes for iron poisoning in clinical practice, and new indications seem to be available based on their antiferroptotic properties. TZDs, a class of typical antidiabetic compounds, have been proven to act as ACSL4 inhibitors to restore GPX4 expression and reduce COX2 expression, and finally prevent lipid peroxidation and ferroptosis to ameliorate intestinal and pulmonary I/R injuries *in vitro* and *in vivo* (Doll et al., 2017; Li Y. et al., 2019; Xu et al., 2020). Furthermore, chelation therapy targeting iron dyshomeostasis has shown promise against neurodegenerative disorders in preclinical studies and clinical trials. Especially, DXZ is currently the only FDA-approved drug for preventing and treating DOX-induced cardiotoxicity in cancer patients (Fang et al., 2019). DFP has been recently used clinically for systemic iron overload dysfunctions and investigated as a conventional or modified treatment in several clinical trials against PD. It has shown reduced iron deposition in the SN and an improvement in motor function, as well as slowed disease progression and relieved neurological symptoms (Devos et al., 2014). Currently, many studies on ferroptosis relevance in neurodegenerative diseases are attempting to use ferroptosis inhibitors, especially iron chelators, to slow disease progression in preclinical animal models, most of which have achieved an potent efficacy (Guo et al., 2016; Rao et al., 2021). Nevertheless, there are still many restrictions and problems associated with chelation therapy. When applied in human clinical trials, iron chelators appears to be far from satisfactory (Grolez et al., 2015). DFP is well tolerated, achieved target engagement (lowering of iron) in patients with neurodegeneration. It seems to somewhat slow disease progression and improve quality of life, but they have not reach significance (Martin-Bastida et al., 2017; Klopstock et al., 2019). Although some of iron chelators, such as DFO, DFP and deferasirox (DFS) are recommended as iron overloaded first-line treatments, they all have their own definite side effects, such as gastrointestinal disorders, hepatotoxicity, nephrotoxicity and visual and auditory neurotoxicity (Vichinsky et al., 2011; Aydinok et al., 2015; Maggio et al., 2020). Furthermore, poor pharmacokinetic properties of DFO and DFS limit their availabilities in the central nervous system, as they are prevented from crossing the blood-brain barrier (BBB) (Sripetchwandee et al., 2016; Crielaard et al., 2017). More appropriate iron chelators that regulate the ferroptotic process need to be further explored and developed for their potential to treat neurodegenerative diseases in preclinical and clinical studies.

Therefore, it is worth noting that nanotechnology has emerged to achieve anticancer effects by improving the pharmacokinetic properties of existing proferroptotic agents or developing novel nano-inducers of ferroptosis. The FDA-approved iron-supplementing agent ferumoxytol (Feraheme) has been proven to have an anti-leukaemia effect *in vitro* and *in vivo* via increasing intracellular iron and ROS production, leading to enhanced oxidative stress and ferroptotic cell death (Trujillo-Alonso et al., 2019). Cisplatin (CDDP)-loaded Fe_3_O_4_/Gd_2_O_3_ hybrid nanoparticles with the conjugation of lactoferrin and RGD dimer (RGD2) (FeGd-HN@Pt@LF/RGD2), which are able to cross the BBB, have been developed for therapy of orthotopic brain tumors based on ferroptosis mechanisms (Shen et al., 2018). In view of the oxidative stress regulation ability of p53 and inducibility of the metal-organic network (MON) on the Fenton reaction, synthesized MON encapsulated with p53 plasmid (MON-p53) can trigger both ferroptosis and apoptosis pathways to synergistically kill cancer cells (Zheng et al., 2017).

Collectively, we have listed FDA-approved synthetic drugs that have been reported to modulate ferroptosis in Table 2 and experimental chemical compounds in Supplementary Tables S1 and Supplementary Tables S2.

**TABLE 2:** FDA-approved synthetic drugs associated with ferroptosis.

Drugs	Functional targets	Impact on ferroptosis	Diseases	Test models	Mechanisms/Effects	Refs
Sulfasalazine (SAS)	System x_c_^-^	Induce	Fibrosarcoma; Breast cancer; Pancreatic cancer	MDA-MB-231, T47D, BT549, MCF7, CFPAC1 cells; PANC1, HT-1080 cells and xenograft mice; Panc02 orthotopic mice	GSH depletion; SLC7A11 downregulation; Lipid peroxidation	Dixon et al. (2012), Zhu et al. (2017), Wang W. et al. (2019), Yu et al. (2019)
Sorafenib	System x_c_^-^	Induce	Hepatocellular carcinoma (HCC)	HepaG2, Hep3B cells; Hepatocytes from HCC patients	Lipid peroxidation; GSH depletion; Increasing ROS level	Lachaier et al. (2014), Sun et al. (2016a), Sun et al. (2016b)
Lanperisone	System x_c_^-^	Induce	K-ras-driven tumors	K-ras-mutant mouse embryonic fibroblasts (MEFs) and xenograft mice	GSH depletion; Increasing ROS level	Shaw et al. (2011)
Glutamate; Glutamine	System x_c_^-^	Induce	Fibrosarcoma	OHSCs, MEFs; HT-1080, HT22 cells; Primary cortical neurons	Inhibiting cystine import; GSH depletion	Dixon et al. (2012), Gao et al. (2015), Bueno et al. (2020)
Statins (fluvastatin, simvastatin, lovastatin acid)	GPX4	Induce	Fibrosarcoma	HT-1080 cells	Downregulating GPX4 level, mevalonate pathway and selenoprotein biosynthesis; Lipid peroxidation	Viswanathan et al. (2017)
Altretamine	GPX4	Induce	DLBCL	OCI-LY3, OCI-LY7, U-2932 cells	GPX4 inactivation; Decreased PC-OOH level; Lipid ROS accumulation	Woo et al. (2015)
Ferumoxytol (Feraheme)	Iron	Induce	Leukemia	19 kinds of leukemia cells; PDXs of leukemia cells	FPN downregulation; Increasing intracellular iron and ROS levels	Trujillo-Alonso et al. (2019)
Salinomycin (ironomycin)	Iron	Induce	Breast cancer	Human breast cancer stem cells (CSCs); PDXs	Iron accumulation and sequestration in lysosomes; Degraded ferritin, the iron storage protein; Increasing ROS and TFRC	Mai et al. (2017)
Ferric ammonium citrate (FAC)	Iron	Induce	Fibrosarcoma	HT-1080 cells	Iron overloading; Oxidative damage; Activating ALOXs; Increasing ROS production	Fang et al. (2018)
Cisplatin	GSH	Induce	NSCLC; Human colon cancer	A549, HCT116 cells	GSH depletion; Increasing ROS level; Inducing both ferroptosis and apoptosis	Guo et al. (2018a), Guo et al. (2018b)
Haloperidol	Sigma 1 receptor	Induce	HCC	HepG2, Huh-7 cells	Increasing cellular levels of HO-1	Bai et al. (2017)
Busulfan	NRF2; GPX4	Induce	—	Oligospermia mice	Downregulating expressions of NRF2, GPX4 and FPN	Zhao et al. (2020)
Siramesine and lapatinib	Iron; FPN	Induce	Breast cancer	MDA-MB-231, SKBR3 cells	Iron and ROS accumulation; Upregulating TF; Downregulating FPN and ferritin	Ma et al. (2017)
Ciclopirox olamine (CPX)	Iron	Inhibit	Neuro-degenerative diseases	HT1080 cells; OHSCs	Removing excess iron; Rescuing cells from erastin-induced ferroptosis	Dixon et al. (2012)
Deferoxamine (DFO)	Iron	Inhibit	Neuro-degenerative diseases; Ageing	HT-1080, Calu-1, BJeLR, PC12, MEF cells; Ageing model mice	Removing excess iron; Rescuing from erastin-induced ferroptosis	Dixon et al. (2012), Chen X. et al. (2020)
Dexrazoxane (DXZ)	Iron	Inhibit	Cardiomyopathy	DOX-induced cardiomyopathy model mice; Acute and chronic I/R model mice	Reducing DOX- cardiomyopathy; Maintaining mitochondrial function	Fang et al. (2019)
Deferiprone (DFP)	Iron	Inhibit	Neuro-degenerative diseases	Primary hippocampal neurons and hippocampus of developing rats and aged mice after general anaesthesia	Iron depletion; Slowing disease progression and improving motor function; Protecting cells against ferroptosis	Wu et al. (2020)
Thiazolidinediones (TZDs) Rosiglitazone pioglitazone troglitazone	ACSL4	Inhibit	Intestinal and pulmonary I/R	ACSL4-knockout MEFs GPX4-knockout mice; Caco-2 cells; Intestinal and pulmonary I/R model mice; Hypoxia-reoxygenation (HR) model A549 cells	Inhibiting ACSL4 and COX2 expression; Restoring GPX4 expression Inhibiting lipid peroxidation and ferroptosis; Improving tissue death	Doll et al. (2017), Li Y. et al. (2019), Xu et al. (2020)
N-acetylcysteine (NAC)	ALOX5	Inhibit	Hemorrhagic stroke	HT-1080 cells; Primary cortical neuronal cultures; ICH model mice and rats	Increasing GSH; Inhibiting active lipids; Neutralizing toxic lipids	Dixon et al. (2012), Karuppagounder et al. (2018)
Zileuton	ALOX5	Inhibit	Neuro-degenerative diseases; Iron overload related diseases	Molt-4, Jurket, HT22, HT-1080 cells	Decreasing ROS production; Rescuing from glutamate oxidative toxicity; Neuroprotective effect	Liu et al. (2015), Probst et al. (2017), Fang et al. (2018)
Vildagliptin; Alogliptin; Linagliptin	DPP4	Inhibit	—	TP53^+/+^ and TP53^−/−^ HCT116 cells and mice	Blocking DPP4-mediated lipid peroxidation; Attenuating the anticancer activity of erastin on TP53^−/−^CRC cells	Xie et al. (2017)
Dopamine	Neuro-transmitter	Inhibit	Neuro-degenerative diseases	HT-22, HT-1080, PANC-1, HEY, HEK293, MEF cells	Reducing GSH depletion; Increasing GPX4, iron accumulation and MDA production; Protecting cells against lipid peroxidation	Wang D. et al. (2016), Tang and Tang, (2019)
Cycloheximide (CHX)	Protein synthesis	Inhibit	Neuro-degenerative diseases	HT1080, Calu-1, BJeLR, MEF cells	Inhibiting protein synthesis; Preventing erastin-induced ferroptosis	Dixon et al. (2012)
Tocotrienols: Vitamin E; α-tocopherols	Lipid peroxidation	Inhibit	Acute lymphocytic chorio-meningitis virus and Leishmania major parasite infections; Hepatic I/R	HT-1080, BJeLR cells; GPX4-deficient T cells and mice; Hepatic I/R model mice	Eliminating peroxygen free radicals; Preventing lipid peroxidation	Yang et al. (2014), Matsushita et al. (2015), Yamada et al. (2020a)
Coenzyme Q10 (CoQ10); Idebenone	Mevalonate pathway	Inhibit	—	Four engineered BJ cell lines (BJeLR, DRD, BJeHLT, BJeH); HT-1080 cells	Inhibiting ROS production	Shimada et al. (2016a)
α-Lipoic acid	Unknown	Inhibit	Alzheimer’s disease (AD)	P301S Tau transgenic mice	Increasing FPN, xCT and GPX4; Decreasing TFRC, iron and ROS generation	Zhang Y. -H. et al. (2018)

### Natural Products and Their Modulation on Ferroptosis

**FIGURE 4:**
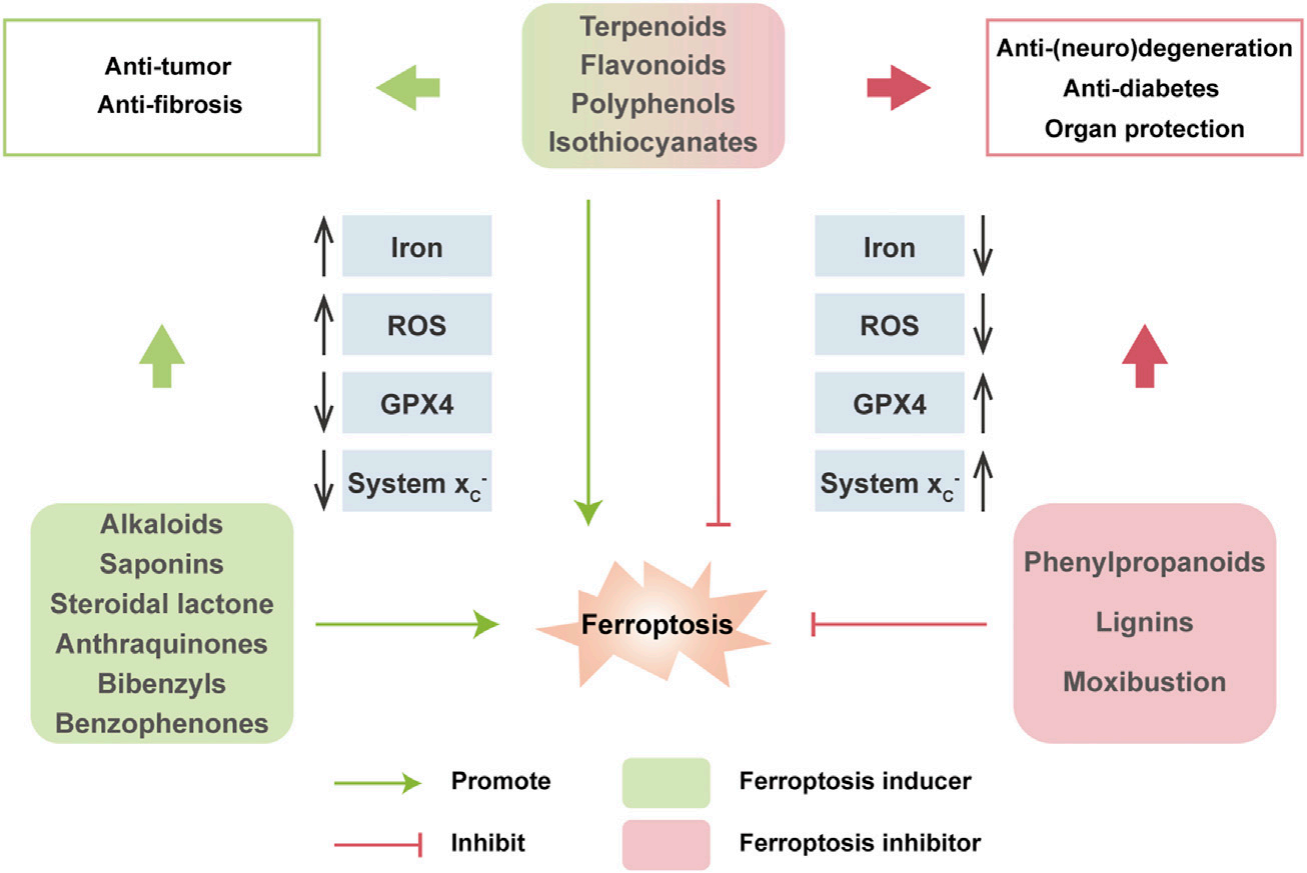
Multiple effects of natural compounds regulating ferroptosis. Alkaloids, saponins, steroidal lactone, anthraquinones, bibenzyls and benzophenones can induce ferroptosis with anticancer and antifibrosis functions. In contrast, phenylpropanoids and ligins alleviate (neuro)degeneration and prevent irreparable organic damage via antagonizing ferroptosis. Interestingly, terpenoids, flavonoids, polyphenols and isothiocyanates have dual effects on ferroptosis which depends on different disease contexts.

Nowadays, natural medicines are usually used alone or, in most cases, in combination with conventional drugs in clinical in the hope of enhancing the efficacies and/or neutralizing the toxicities of chemical drugs. In addition to clinical (adjuvant) medicines, natural products are widely consumed as supplements in alternative strategies (Shu-Feng Zhou et al., 2007; Kibble et al., 2015). Growing evidence has uncovered the solid regulatory properties of ingredients with bioactive functions on ferroptosis (Figure 4). Hence, we focus on natural compounds that show a pharmacological potential for ferroptosis-associated (adjuvant) therapy.

#### Terpenoids

Terpenoids are important sources for the research of natural products and the development of modern drugs with a great deal of pharmacological functions such as inflammation, neurodegeneration and cancers. **Artemisinin** and its derivatives (**Artemisinins**, including **artesunate**, **artemether**, **dihydroartemisinin**, etc.) are sesquiterpene lactone ingredients derived from *Artemisia annua* L., and are well known globally for their antimalarial bioactivity (Dondorp et al., 2005). Beyond the antimalarial efficacy, artemisinins have been well documented to be potently effective in diverse diseases *in vitro* and *in vivo*. Artemisinins have been extensively demonstrated to induce ferroptosis for cancer treatment through various mechanisms, including regulating iron-related genes, promoting ROS production, depleting intracellular GSH, and increasing intracellular iron levels and activating ferritinophagy. Artesunate (ART), a water-soluble derivative of artemisinin, has been approved as a ferroptosis inducer to selectively promote ROS- and lysosomal iron-dependent cell death in KRAS-transformed PDAC cells, which are highly resistant to apoptosis. Of note, ART exerts no effect on nonneoplastic human pancreatic ductal epithelial cells (Eling et al., 2015). Surprisingly, ART induces ferroptosis via the accumulation of iron, leading to elevated lipid peroxides and reduced antioxidant capacity in several other cancers, such as ovarian cancer, head and neck cancer and Burkitt’s lymphoma (Greenshields et al., 2017; Roh et al., 2017; Wang N. et al., 2019). ART has undergone several clinical trials against various cancers, including metastatic breast cancer and cervical intraepithelial neoplasia, and has been proven to be safe and well-tolerated in patients (von Hagens et al., 2017). Dihydroartemisinin (DHA), one of the semisynthetic derivatives of artemisinin, can trigger ferroptosis to directly kill cancer cells or synergistically sensitize the efficacy of chemotherapeutic agents via multiple functions, including autophagic degradation of ferritin, direct binding to free iron and disturbing iron homeostasis in a cohort of cancer cells (Chan et al., 2013; Du et al., 2019; Chen G. -Q. et al., 2020). Interestingly, DHA activates the feedback pathway of ferroptosis in glioma cells. That is, under endoplasmic reticulum stress induced by DHA, the subsequent increase in GPX4 expression counteracts lipid peroxidation-mediated ferroptosis (Chen et al., 2019). In addition to antineoplastic activities, artemisinins also exhibit hepatoprotective effects via the execution of ferroptosis. ART and artemether have been proven to trigger p53-dependent ferroptosis by promoting the accumulation of iron and lipid peroxides, which can inactivate hepatic stellate cells (HSCs) and ameliorate hepatic fibrosis (Wang L. et al., 2019; Kong et al., 2019). Danshen (*Salvia miltiorrhiza*) is a commonly used traditional Chinese medicinal herb for the clinical therapy of many diseases. However, two diterpenoid compounds, **dihydroisotanshinone I** (DT) and **cryptotanshinone** isolated from Danshen show opposite functions in cancer cells. DT treatment significantly induces ferroptosis to suppress breast carcinoma through downregulating GPX4 protein expression in cell and xenograft models (Lin et al., 2019). In contrast, **cryptotanshinone** definitely blocks erastin-induced ferroptosis in PDAC cells upon antagonizing STAT3, a positive modulator of ferroptosis (Gao et al., 2018). **Glycyrrhizin** (GLY) and **magnesium isoglycyrrhizinate** (MgIG) are natural ingredients extracted from the root of *Glycyrrhiza* with neuroprotective activity. Nevertheless, current studies have demonstrated that GLY and MgIG protect hepatic function through different modulations of ferroptosis in different subtypes of hepatic cells. MgIG remarkably induces HSC ferroptosis through iron accumulation and ROS production, and finally ameliorates CCl_4_-induced hepatic fibrosis where HO-1 plays an important role (Sui et al., 2018). Yet, GLY, a high mobility group protein B1 (HMGB1) inhibitor, has been shown to reduce the degree of ferroptosis of hepatocytes during acute liver failure (ALF) via activating NRF2/HO-1/HMGB1 pathway to antagonize oxidative stress (Wang Y. et al., 2019).

#### Flavonoids

Flavonoids are extensively distributed in the plant kingdom, with multifaceted pharmacological properties and diversified molecular structures. Due to powerful free radical scavenging and antioxidant action, many flavonoids have been reported to be potential ferroptosis inhibitors. **Baicalein** (also termed 5,6,7-trihydroxyflavone) is a flavonoid originally isolated from the whole root of *Scutellaria baicalensis*, and has been shown to be effective against cancer, bacterial infections, organ dysfunctions and oxidative stress diseases. Before ferroptosis was formally coined, solid evidence has revealed that baicalein acted as a strong iron chelating agent, which inhibited the Fenton reaction and lipid peroxidation by forming an iron-baicalein complex (Perez et al., 2009). It is reasonable to speculate that baicalein plays its pharmacological role through the above ferroptosis-associated mechanisms. Emerging studies have shown that baicalein is a selective inhibitor of ALOX12/15, which in turn strongly downregulates prostaglandin-endoperoxide synthase 2 (PTGS2) and upregulates GPX4, and significantly suppresses ferroptosis to exhibit a neuroprotective role in post-traumatic epileptic seizures (Li Q. et al., 2019). Furthermore, as a natural ferroptosis inhibitor, baicalein limits iron production, GSH depletion, GPX4 degradation and lipid peroxidation to reverse erastin- or RSL3-induced ferroptosis in pancreatic cancer cells (Xie et al., 2016b; Probst et al., 2017). **Quercetin** (3,30,40,5,7-pentahydroxyflavone) is one of the most widely distributed flavonoids in edible and medicinal plants, such as onion, tea and ginkgo biloba, and displays a variety of biological activities, especially antioxidative capacity. A recent study has demonstrated that type 2 diabetes (T2DM) molding depletes GSH, downregulates GPX4, and induces oxidative stress in pancreatic β cells (PBCs), indicating that ferroptosis contributes to the loss and dysfunction of PBCs. Indeed, quercetin protects PBCs from pancreatic iron deposition to alleviate ferroptosis in T2DM (Li D. et al., 2020). Moreover, both quercetin and its metabolite quercetin Diels-Alder anti-dimer (QDAD) prevent bone marrow-derived mesenchymal stem cells (bmMSCs) from erastin-induced ferroptosis possibly through the antioxidant pathway, which in turn converts quercetin into QDAD, an inferior ferroptosis inhibitor and antioxidant (Li X. et al., 2020). Despite that flavonoids discussed above are usually approved as strong antioxidants, there are some other flavonoids functioning as ferroptosis inducers, including **amentoflavone** (AF), a polyphenolic flavonoid widely found in *Selaginella* and **typhaneoside** (TYP), a major flavonoid in the extract of *Pollen Typhae*. Both AF and TYP trigger ferroptosis in an autophagy-dependent manner via AMPK signaling, contributing to ferritin degradation, ROS accumulation and ultimately ferroptotic cell death in acute myeloid leukemia (AML) and glioma (Zhu et al., 2019; Chen Y. et al., 2020).

#### Polyphenols

Phenolic compounds isolated from plants are known as a fighter against various diseases related to oxidative stress, such as cancer, inflammation, obesity, organic damage and neuronal disorders (Curti et al., 2017). **Curcumin**, a polyphenol extracted from the rhizome of turmeric and other plants of the family Zingiberaceae, is a powerful antioxidant compound with a great deal of pharmacological activities. Notably, curcumin has been demonstrated to enhance antioxidative capacity as a ferroptosis inhibitor. In renal tubular cells, curcumin inhibits myoglobin (Mb)-induced ferroptosis and ameliorates renal injury associated with rhabdomyolysis by inhibiting the TLR4/NF-κB axis and activating the cytoprotective enzyme HO-1 (Guerrero-Hue et al., 2019). **Proanthocyanidins** (PACs), widely found in plants in nature, are potent free radical scavengers. PAC treatment significantly decreases the levels of iron, ACSL4, and ALOX15B, while increasing the levels of GSH, GPX4, NRF2, and HO-1 in the traumatic spinal cord, eventually rescues ferroptosis-induced injuries and improves the locomotive function of spinal cord injury (SCI) mice (Zhou et al., 2020). Similarly, **Epigallocatechin gallate** (EGCG), the most abundant catechin in tea, is a polyphenol with various pharmacological benefits. Both curcumin and EGCG have been demonstrated to prevent ferroptosis against iron-induced oxidative damage in mouse MIN6 pancreatic β cells through neutralizing iron, reducing GSH consumption as well as preventing GPX4 inactivation (Kose et al., 2019). Beyond the organ protection effect, the anticancer efficacy of polyphenols has been well established by accumulated clinical experience, where ferroptosis is believed to be involved. EGCG is a direct inhibitor of heat shock 70 kDa protein 5 (HSPA5), which has been proven to negatively regulate ferroptosis. Further research shows that EGCG can prevent the formation of the anti-ferroptotic HSPA5-GPX4 complex and subsequent lipid peroxidation, which enhances gemcitabine sensitivity by disinhibiting ferroptosis in PDAC *in vitro* and *in vivo* (Zhu et al., 2017). Interestingly, EGCG either prevents ferroptosis in normal pancreatic β cells, or induces ferroptosis in cancerous PDAC cells, implying context-dependent bidirectional regulation and special pancreatic for pancreas protection of EGCG.

#### Isothiocyanates

Isothiocyanates (ITCs) are abundant in cruciferous vegetables, exhibiting chemopreventive and chemotherapeutic properties to tackle many types of diseases. Two ITCs, **phenylethyl isothiocyanate** (PEITC) and **sulforaphane** have been employed in small human clinical trials against various diseases ranging from cancer to autism, either within cruciferous food or as single agents (Palliyaguru et al., 2018). PEITC is a dietary anticancer compound with pharmacokinetic behaviors of rapid absorption and high bioavailability (Ji et al., 2005). It has been documented that PEITC triggers multiple forms of cell death, namely ferroptosis, apoptosis, and autophagy in both murine and human osteosarcoma models. PEITC drives oxidative stress as a consequence of GSH depletion-induced ROS generation and lipid peroxidation. ROS generation is proven to be the major cause of PEITC-induced cell death, accompanied by altered iron metabolism and activation of the MAPK signaling pathway (Lv H. -H. et al., 2020; Lv H. et al., 2020). However, another ITC, sulforaphane, acting as a ferroptosis inhibitor, reduces busulfan-induced damage to prevent oligospermia by activating the upregulation of GPX4 and FPN1 protein expression (Zhao et al., 2020).

#### Alkaloids

Alkaloids are one type of important active component in Chinese herbal medicine and display a broad range of biological properties, such as neurogenesis promotion, pro-oxidant activities, and anti-infection. **Trigonelline** is an alkaloid derived from a traditional Chinese herbal, *Trigonella foenum-graecum L*., present in coffee and fenugreek seeds in considerable amounts (Sun et al., 2016a). Trigonelline has been believed to be of great benefits in the prevention and treatment of diabetes and central nervous system disorders (Zhou et al., 2012). Recent studies have revealed that trigonelline exerts antitumor efficiency as a pharmacological inhibitor of NRF2. The inhibition of NRF2 by trigonelline enhances the anticancer activity of classical ferroptosis inducers, such as erastin, sorafenib and RSL3, and sensitizes chemoresistant cancer cells to ferroptosis both *in vitro* and *in vivo* models of HCC and HNC (Sun et al., 2016a; Shin et al., 2018). **Solasonine** isolated from *Solanum melongena* has various pharmacological benefits. Solasonine has been proven to inhibit the growth and migration of HCC cells. Detailed studies elucidate that solasonine increases lipid ROS levels via GPX4-induced destruction of the GSH redox system, leading to the promotion of ferroptosis (Jin et al., 2020).

#### Saponins

Saponins are the major active ingredients of many Chinese herbal medicines, such as ginseng, licorice, and bupleurum, processing valuable biological activities including antipyretic, anticancer properties, as well as immunoregulation. **Ruscogenin**, is initially extracted from *Ruscus aculeatus* and is a main component of *Radix Ophiopogon japonicas*, with a wide range of biological functions (Song et al., 2020). Acting as a promising ferroptosis inducer, ruscogenin is found to increase ferrous iron and aberrant ROS production and regulate the levels of TF and FPN, which triggers ferroptosis against pancreatic cancer *in vitro* and *in vivo*. **Ardisiacrispin B**, a natural oleanane-type tritepene saponin isolated from the fruit of *Ardisia kivuensis* Taton (Myrsinaceae), displays a significant cytotoxic effect in a panel of 9 cancer cell lines (including various sensitive and drug-resistant phenotypes) depending on ROS-mediated ferroptosis (Mbaveng et al., 2018a).

#### Others

Actually, many kinds of natural compounds serve as potential modulators of ferroptosis. **Withaferin A** (WA), a major constituent of *Withania somnifera* extract, has been well documented to provide pharmacological benefits. WA can obviously induce ferroptosis to suppress high-risk neuroblastoma via a double-edged mechanism. On the one hand, WA decreases the protein level and activity of GPX4, which resembles the canonical ferroptosis-inducing pathway. On the other hand, WA induces excessive lipid peroxidation by increasing the labile Fe (II) pool upon HO-1 activation through direct targeting of Keap1, which represents for a novel noncanonical ferroptosis pathway (Hassannia et al., 2018). **Erianin**, a natural product isolated from a famous TCM, *Dendrobium chrysotoxum Lindl*, has been reported to exert anticancer effects on several cancer types. Erianin has shown to inhibit the growth and migration of lung cancer cells via Ca^2+^/CaM-mediated ferroptosis, accompanied by ROS accumulation, GSH depletion and lipid peroxidation (Chen P. et al., 2020). *Cullen corylifolium* is a famous TCM known as “Buguzhi” and listed officially in Chinese pharmcopoeia, containing various phytochemical compounds such as flavonoids and coumarines. The extracts have been approved to be effective for its anti-inflammatory, antioxidation, neuroprotection properties. Compound 3 (**psoralidin**) isolated from *Cullen corylifolium* displays tremendous inhibitory affinity for ALOX and Keap1-NRF2 protein-protein interactions, two important ferroptosis-related targets, and shows anti-ferroptotic activities on erastin-treated hippocampal HT22 cells (Yaseen et al., 2021). **Nordihydroguaiaretic acid** (NDGA), a lignin contained in *Larrea tridentata*, is a potent antioxidant. As a pan-ALOX inhibitor, NDGA effectively prevented lipid peroxidation, ROS generation and ferroptosis induced by proferroptotic reagents in BT-474, AML12 and acute lymphoblastic leukemia (ALL) cells (Hangauer et al., 2017; Probst et al., 2017; Fang et al., 2018).

#### Cooperation of Multiple Natural Compounds

In addition to single compound, cooperation of multiple (two or more) natural products or direct extracts of herbals and TCM formulations that are rich in various compounds, actually exhibits pro- or anti-ferroptosis pharmaco-activities. **Cotylenin A** (CN-A, plant growth regulator) combined with PEITC in the treatment of pancreatic cancer can synergistically induce ROS production and lead to cell death mainly due to the induction of ferroptosis, while CN-A or PEITC alone fails to induce ferroptosis (Kasukabe et al., 2016). ***Betula etnensis* Raf** (Birch Etna) belongs to the Betulaceae family, which contains multiple antineoplastic ingredients in bark, including polyphenols, terpenoids, betulin, betulinic acid, and ursolic acid. Methanolic extract of *B. etnensis* Raf. promotes ROS production and an oxidative cellular microenvironment, which results in the death of colon cancer cells dependent on ferroptosis mediated by HO-1 hyper-expression and lipid peroxidation (Malfa et al., 2019). **Brown rice** is known as an antioxidant-rich food with a high level of vitamin E. Methanol extracts of brown rice have been reported to be a ferroptosis inhibitor and protect cells against lipid peroxidation via compensating for GPX4 loss, suggesting that brown rice is useful for the pathologies of vascular diseases driven by lipid peroxidation (Sakai et al., 2017). **Naotaifang** is a compound traditional Chinese herbal medicine consisting of four herbs, namely, *Radix Astragali* (Huangqi), *Rhizoma chuanxiong* (Chuangxiong), *Pheretima* (Dilong) and *Bombyx batryticatus* (Jiangcan). Its extract (NTE) has clinical benefits for the neurological improvement of patients with acute cerebral ischemia. NTE can ameliorate neuronal ferroptosis in MCAO rats through the TFRC/DMT1 and SCL7A11/GPX4 pathways (Lan et al., 2020).

#### Clinical Therapeutic Potential of Natural Products Targeting Ferroptosis

Iron homeostasis, lipid peroxidation and GSH metabolism are three mainstays in the modulation of ferroptosis, and are supposed to be promising in ferroptosis-associated diseases. Based on physiopathological characteristics, proferroptotic compounds are potent anticancer candidates, whereas anti-ferroptotic agents are organo-protective therapies. Apart from chemical compounds, natural products, including TCM resources, which are rich in nature, provide abundant bioactive ingredients that modulate ferroptosis. Indeed, preclinical and clinical studies have demonstrated that many natural compounds with ferroptotic activity exhibit definite regulatory effects on the cyst(e)ine/GSH/GPX4 antioxidative axis (Figure 4). Of note, many of them, including artemisinin and its derivatives, β-elemene, SFN, baicalein, kaempferol, quercetin, curcumin and EGCG, have been evaluated in extensive clinical trials for various pharmacological effects (Wang Y. et al., 2017; Luo et al., 2019; Parvez and Rishi, 2019). Except for antimalarial effects, artemisinin and its derivatives seem to be good proferroptotic candidates and have been evidenced for their immune surveillance to inhibit carcinogenesis, tumor progression and metastasis (Kiani et al., 2020). For cancer therapy, a large body of studies have indicated that artemisinin and its derivatives trigger ferroptosis through overproducing ROS and regulating the x_c_^-^/GPX4 system axis in various cancer cells, which has undergone several clinical trials and is proven to be safe and well-tolerated in patients with metastatic breast cancer or advanced-stage solid tumors (von Hagens et al., 2017). Additionally, β-elemene (a sesquiterpene) injection has been approved recently as adjuvant therapy by China’s State Food and Drug Administration (SFDA) for lung cancer (Wang et al., 2012). Mechanistically, combinative treatment with β-elemene and cetuximab induces ferroptosis and suppresses CRC migration by regulating EMT through both GPX4-dependent and-independent pathways (Chen P. et al., 2020). Two ITCs, PEITC and sulforaphane protect cells from ferroptosis via limiting iron accumulation and preventing GPX4 degradation and have been investigated in clinical trials against various diseases ranging from cancer to autism, either alone or in combination with other drugs (Palliyaguru et al., 2018). In addition to proferroptotic cancer treatment, bioactive ferroptosis inhibitors, which are usually antioxidants that restore GSH/GPX4 levels, have also been clinically investigated to treat other diseases. EGCG, a famous antioxidant has been evaluated in numerous clinical trials for various disorders for decades, such as metabolic syndromes, neurodegenerative diseases and cardiovascular diseases. EGCG has been proven to protect pancreatic β cells against iron toxicity and erastin-induced ferroptosis through neutralizing iron and preventing GPX4 inactivation (Kose et al., 2019). Moreover, EGCG can also act as an iron chelator to regulate the TFRC/FTH expression and to prevent GPX4 activation, resulting in the inhibition of ferroptosis (Md Nesran et al., 2020). In addition to its antioxidative properties, EGCG is evidenced by clinical experience as an anticancer agent that modulates GPX4 levels to promote ferroptosis (Zhu et al., 2017). Owing to the context-dependent regulation of ferroptosis based on GPX4, EGCG has been recognized as a promising effective ferroptosis-modulating compound for clinical application or herbal supplementation.

Many natural compounds that are already in clinical use or have a strong potential for clinical translation are known to promote or block ferroptosis. Compared with classical ferroptosis regulators, TCM has the characteristics of many regulatory targets, stable structure, high safety, low cost and easy availability. However, it remains to be determined whether their biological activities rely on ferroptosis-regulating properties. The lack of specific biomarkers and molecular mechanisms for ferroptosis blocks the clinical transformation of novel ferroptosis-associated treatments mediated by natural product candidates. Due to starting late and accumulating less, more studies should be conducted to avoid side effects and to develop individualized and precise therapy.

Apart from the above discussed representative compounds, more natural products that have been reported to be able to induce/inhibit ferroptosis are classified and summarized in Tables 3, 4.

**TABLE 3:** Natural products induced ferroptosis.

Classification	Compounds	Functional targets	Diseases	Test models	Mechanisms/Effects	Refs
Terpenoids	Artemisinin	Iron	Osteosarcoma	D-17 cells	Decreasing iron levels	Isani et al. (2019)
	Artesunate	NRF2; p62; FTH1; Activating transcript-tion factor 4 (ATF4)	Head and neck cancer (HNC); Pancreatic cancer; Ovarian cancer; Burkitt’s lymphoma; Liver fibrosis	Several HNC/PDAC/ovarian cancer cells and xenograft mice; DAUDI and CA-46 cells and xenograft mice; Mouse HSCs; LX2 cells	Decreasing GSH level; Increasing iron and lipid ROS levels; Increasing GRP78 levels; Inducing ATF4-CHOP-CHAC1 pathway	Eling et al. (2015), Greenshields et al. (2017), Roh et al. (2017), Wang N. et al. (2019), Wang K. et al. (2019), Kong et al. (2019)
	Dihydroartemisinin (DHA)	FTH1; GPX4	Several types of cancers, including glioma, head and neck carcinoma; Acute myeloid leukemia	Dozens of multiple cancer cells; Human umbilical vein endothelial cells (HUVECs), MEFs; U251, U373, HL60, H292 xenograft mice; Patient-derived glioma cells	Inducing lysosomal degradation of ferritin; Inducing iron and ROS accumulation; Inhibiting GPX4 expression; Activating the feedback path of ferroptosis	Chan et al. (2013), Lin et al. (2016), Chen et al. (2019), Du et al. (2019), Chen G. -Q. et al. (2020)
	Artemether	Iron	Liver fibrosis	HSC-T6 cells; CCl_4_-induced hepatic fibrosis model mice	Promoting accumulation of iron and lipid peroxides; Inducing p53-dependent ferroptosis of HSC; Ameliorating CCl_4_-induced hepatic fibrosis	Wang L. et al. (2019)
	Dihydro-isotanshinone I (DT)	GPX4	Breast cancer	MDA-MB-231 cells; MCF-7 cells and xenograft mice; Patients	Reducing GSH/GSSG ratio and GPX4 activity; Inducing apoptosis and ferroptosis	Lin et al. (2019)
	Magnesium isoglycyrrhizinate (MgIG)	HO-1	Liver fibrosis	CCl_4_-induced liver fibrosis model rats; HSC-T6 cells	Increasing HO-1, TF, TFRC expression and nuclear abundance; Reducing GSH level and FPN expression; Increasing levels of ROS, iron and lipid peroxides	Sui et al. (2018)
	Ferroptocide, a compound from *pleuromutilin*	Thioredoxin	Many cancer types	A dozen of cancer cell lines and primary cancer cells	Inhibiting thioredoxin; Inducing lipid ROS accumulation	Llabani et al. (2019)
	β-elemene	Unknown	Colorectal cancer	HCT116, LOVO, Caco-2 cells; Orthotopic HCT116 mice	Inducing iron-mediated ROS accumulation, GSH depletion, lipid peroxidation; Upregulating HO-1 and TF and downregulating GPX4, FTH1, GLS, SLC7A11, SLC40A1	Chen et al. (2020e)
	Pseudolaric acid B (PAB)	p53; TFRC; NOX4; xCT	Glioblastoma	SHG-44, U87, U251 cells; C6 cells and xenograft mice	Upregulating TFRC and NOX4; Increasing ferrous and lipid peroxidation; Inducing GSH exhaustion by xCT inhibition	Wang Z. et al. (2018)
Flavonoids	Amentoflavone	FTH1; AMPK/mTOR signaling	Glioblastoma	Normal human astrocytes; U373 cells; U251 cells and xenograft mice	Increasing intracellular levels of iron, MDA and lipid ROS; Reducing levels of GSH, FTH1 and the mitochondrial membrane potential	Chen et al. (2020c)
	Typhaneoside (TYP)	AMPK/mTOR signaling	Acute myeloid leukemia	Kas-1, NB4, K562, 293T cells HL60 cells and xenograft mice	Triggering autophagy by activating AMPK signaling; Inducing ferritin degradation, ROS accumulation and ferroptosis	Zhu et al. (2019)
Polyphenols	Epigallocatechin gallate (EGCG)	HSPA5	Pancreatic cancer	PANC1, CFPAC1 cells	Inhibiting GPX4 activity; Enhancing erastin-induced MDA production and ferroptosis	Zhu et al. (2017)
	Gallic acid	GPX4	Cervical cancer; Lung cancer Neuroblastoma; Breast cancer; Melanoma	HeLa, H446, SH-SY5Y, MDA-MB-231, MCF10A, A375 cells; Human dermal fibroblasts (HDF)	Decreasing GPX4 activity; Promoting ROS generation and lipid peroxidation	Tang and Cheung, (2019), Khorsandi et al. (2020)
Iso-thiocyanates	β-Phenethyl isothiocyanate (PEITC)	MAPK signaling pathway	Osteosarcoma	K7M2, U-2 OS, MG-63, 143B cells; MNNG/HOS cells and xenograft mice; Orthotopic osteosarcoma mice	Triggering ROS accumulation; Inducing GSH depletion	Lv H. -H. et al. (2020), Lv H. et al. (2020)
Alkaloids	Trigonelline	NRF2	Hepatocellular carcinoma; (HCC); HNC	HepG2, SNU-182, Hep3B cells; Hepa1-6 cells and xenograft mice; Several HNC cells; Cisplatin-resistant HNC xenograft mice	Blocking NRF2; Inducing GSH depletion and ROS production; Increasing iron level	Sun et al. (2016a), Shin et al. (2018)
	Piperlongumine (PL)	GSH; GSTP1; Thioredoxin reductase (TrxR)	Pancreatic cancer	MIAPaCa-2, PANC-1, CFPAC-1, BxPC-3 cells	Increasing ROS level; Decreasing GSH level	Yamaguchi et al. (2018)
	Ungeremine	Unknown	Breast cancer; Leukemia; Glioblastoma; Colon cancer; Liver cancer	Several cell models including sensitive and resistant counterparts	Increasing ROS production; Inducing apoptosis, necrosis and ferroptosis	Mbaveng et al. (2019)
	Solaso​​nine	GPX4	HCC	HepRG cells; HepG2 cells and xenograft mice	Inhibiting GPX4 and GSS expressions; Increasing lipid ROS	Jin et al. (2020)
Saponins	Ruscogenin	TF; FPN	Pancreatic cancer	SW 1990, PANC-1, AsPC-1, HPDE6-C7 cells; BxPC-3 cells and xenograft mice	Increasing ferrous irons and ROS production	Song et al. (2020)
	Ardisiacrispin B	Iron	Leukemia; Breast cancer; Colon cancer; Glioblastoma	Several cell models including sensitive and their resistant counterparts	Increasing ROS production; Inducing ferroptosis and apoptosis	Mbaveng et al. (2018a)
	Albiziabioside A derivative, Compounds D13	p53	Colon cancer	HCT116 cells and xenograft mice	Activating p53; Reducing mitochondrial membrane potential and GPX4 expression; Inducing ROS production and lipid peroxidation	Wei et al. (2018)
	N-acetylglycoside of oleanolic acid (aridanin)	Unknown	Many kinds of cancer	18 cancer cell lines with sensitive and drug-resistant phenotypes; Metastasizing B16/F10, HepG2, AML12 cells	Increasing ROS levels and mitochondrial membrane potential (MMP) breakdown	Mbaveng et al. (2020)
Steroidal lactone	Withaferin A	GPX4; NRF2	Neuroblastoma	A dozen of neuroblastoma cells; IMR-32 cells and xenograft mice	Inducing lipid peroxidation; Reducing GPX4 activity; Activating HO-1	Hassannia et al. (2018)
Anthra-quinones	Physcion 8-O-β-glucopyranoside	GLS2	Gastric cancer	MKN-45 cells; MGC-803 cells and xenograft mice	Increasing levels of ROS, Fe^2+^ and MDA; Inducing ferroptosis via miR-103a-3p/GLS2 axis	Niu et al. (2019)
Bibenzyls	Erianin	Ca^2+^/CaM signaling	Lung caner	H1299 cells; H460 cells and orthotopic mice	Inducing ROS generation, lipid peroxidation and GSH exhaustion	Chen et al. (2020d)
Benzo- phenones	Epunctanone	Unknown	Many kinds of cancer	9 cancer cell lines including sensitive and drug-resistant cell lines	Increasing ROS levels and MMP breakdown	Mbaveng et al. (2018b)
Multiple	Cotylenin A+ PEITC	Unknown	Pancreatic cancer	(resistant)PANC-1, MIAPaCa-2 cells	Inducing ROS production	Kasukabe et al. (2016)
	Methanolic extract of *Betula etnensis* Raf. bark	HO-1	Colon cancer	Caco-2 cells	Increasing ROS and lipid peroxidation; Reducing HO-1 activity	Malfa et al. (2019)
	Actinidia chinensis Planch (ACP)	EMT; GPX4; xCT	Gastric cancer	HGC-27 cells and zebrafish xenografts	Inhibiting GPX4 and xCT proteins; Inducing ROS accumulation	Gao et al. (2020)
	Bromelain	ACSL4	Colorectal cancer	Caco-2, NCI-H508, HCT116, DLD1, G13D, G12D cells; DSS-treated KRAS mutant mice	Increasing ACSL4 level, lipid biosynthesis and fatty acid degradation	Park et al. (2018)

**TABLE 4:** Natural products inhibited ferroptosis.

Classification	Compounds	Functional targets	Diseases	Test models	Mechanisms/Effects	Refs
Terpenoids	Cryptotanshinone	STAT3	Pancreatic cancer	PANC-1, CFPAC1 cells	Inhibiting STAT3; Blocking erastin-induced ferroptosis	Gao et al. (2018)
	Glycyrrhizin (GLY)	HMGB1	Acute liver failure (ALF)	L02 hepatocytes; ALF mice	Reducing levels of Fe^2+^, MDA, ROS and lactic dehydrogenase (LDH); Increasing NRF2, HO-1 GPX4 and GSH levels; Hepatoprotective effect	Wang Y. et al. (2019)
	Bakuchiol and 3-hydroxybakuchiol, isolated from *Cullen corylifolium*	Unknow	Ferroptosis-related diseases	HT22 cells	Inhibiting erastin-induced ferroptosis	Yaseen et al. (2021)
Flavonoids	Baicalein	ALOX12; ALOX15; PTGS2; GPX4	Post-traumatic epilepsy (PTE); Ferroptosis-induced diseases	HT22, PANC-1, BxPc3, Molt-4, Jurkat cells; FeCl_3_-induced PTE mice	Decreasing ROS, 4-HNE, PTGS2 and ALOX12/15; Inhibiting GSH depletion and increasing GPX4 expression	(Xie et al., 2016b; Probst et al., 2017; Li Q. et al., 2019)
	Quercetin; quercetin Diels-Alder anti-dimer (QDAD)	Antioxidant pathway	T2DM; Degenerative diseases	Bone marrow-derived mesenchymal stem cells (BMSCs); T2DM mice	Lowering iron level; Upregulating GSH, GPX4 and antioxidant pathway	(Li D. et al., 2020; Li X. et al., 2020)
	Puerarin	NOX4; GPX4; FTH1	Heart failure	H9c2 cells; Heart failure model rats	Inhibiting NOX4 expression; Increasing GPX4 and FTH1 expression	Liu et al. (2018)
	Morachalcone D, Morachalcone E	GPX4; NRF2; SLC7A11	Neuro-degenerative diseases	HT22 cells	Preventing ROS production, GSH depletion and iron accumulation; Increasing SLC7A11, GPX4, NRF2 and HO-1 levels; Neuroprotective effect	Wen et al. (2020)
	Butein; (s) -Butin	Antioxidant pathway	Degenerative diseases	BMSCs	Making cells resistant to ferroptosis	Liu J. et al. (2020)
	Sterubin	Unknown	Neuro-degenerative diseases	HT22, BV2, MC65 cells	Activating NRF2/ATF4 Signaling; Protecting against oxytosis/ferroptosis by maintaining GSH levels	Fischer et al. (2019), Maher et al. (2020)
	7-O-Esters of taxifolin: 7-O-cinnamoyl-taxifolin; 7-O-feruloyl-taxifolin	GSH; NRF2	Alzheime’s disease (AD)	BV-2, HT22 cells; AD model mice	Scavenging free radical; Maintaining GSH under stress conditions; Increasing NRF2 level; Neuroprotective effect	Gunesch et al. (2020)
	6 flavonoids isolated from *Cullen corylifolium*	Unknow	Ferroptosis-related diseases	HT22 cells	Inhibiting erastin-induced ferroptosis	Yaseen et al. (2021)
	Kaempferol, Kaempferide	ARE; ROS	Neuro-degenerative diseases	HT22 cells	Inducing ARE activity; Suppressing intracellular ROS and mitochondrial superoxide anion production	Takashima et al. (2019)
Polyphenols	Proanthocyanidin (PACs)	Unknown	Spinal cord injury (SCI)	SCI mice	Reducing iron, ACSL4 and ALOXs levels and oxidative stress; Increasing GSH, GPX4, NRF2 and HO-1 levels	Zhou et al. (2020)
	Curcumin	Iron; GPX4	Acute kidney injury; Pancreatic damage	HK-2, MIN6 cells; Proximal murine tubular epithelial cells (MCTs) Rhabdomyolysis mice	Activating HO-1; Reducing inflammation and oxidative stress; Inhibiting TLR4/NF-κB axis; Decreasing iron level, GPX4 inactivation, GSH depletion, and lipid peroxidation	Guerrero-Hue et al. (2019), Kose et al. (2019)
	Epigallocatechin gallate (EGCG)	Iron; GPX4	Pancreatic damage	Mouse MIN6 pancreatic β cells	Decreasing iron level, GPX4 inactivation, GSH depletion, and lipid peroxidation	Kose et al. (2019)
Iso-thiocyanates	Sulforaphane	NRF2	Oligospermia	Oligospermia mice	NRF2 agonist; Up-regulating GPX4 and FPN protein expression	Zhao et al. (2020)
Phenyl-propanoids	Psoralidin	ALOX5; Keap1-NRF2 pathway	Ferroptosis-related diseases	HT22 cells	Inhibiting ALOX5 and Keap1-NRF2 protein-protein interactions; Neuroprotective effect	Yaseen et al. (2021)
	Artepillin C	ROS	Neuro-degenerative diseases	HT22 cells	Reducing intracellular ROS and mitochondrial superoxide anion production	Takashima et al. (2019)
Lignins	Nordi-hydroguaiaretic acid (NDGA)	ALOX5	Iron overload related diseases	BT474, HT-1080, Molt-4, Jurkat, AML12 cells	Reversing ferroptosis caused by GPX4 inhibition; Protecting cells from iron overload, lipid peroxidation, ROS generation	Hangauer et al. (2017), Probst et al. (2017), Fang et al. (2018)
Multiple	Brown Rice extract	GPX4	Vascular endothelial disorders	HUVECs	Compensating GPX4 loss and preventing LDH release; Decreasing lipid peroxides	Sakai et al. (2017)
	Naotaifang Extract	SLC11A2 (DMT1); Iron; GPX4	Acute brain injury	MCAO rats	Decreasing TFRC and SLC11A2 expressions; Reducing ROS, MDA and iron accumulations; Increasing SLC7A11, GPX4 expressions and GSH level; Neuroprotective effect	Lan et al. (2020)
	Moxibustion	GPX4; FTH1	Parkinson’s disease (PD)	PD model rats	Upregulating GPX4 and FTH1 levels; Neuroprotective effect	Lu et al. (2019)

## Conclusion and Perspectives

Nowadays, numerous studies have shown an ever-growing enthusiasm in exploring the role of ferroptosis in the occurrence and progression of various human diseases. A better understanding of the regulatory mechanisms underlying ferroptosis occurrence and susceptibility will facilitate the identification of better targets for the treatment of diversified diseases and the optimization of clinical prevention, diagnostics, prognostics and treatment. Therefore, the activation of ferroptosis-related sites can induce not only the death of ordinary tumor cells, but also the death of drug-resistant tumor cells. In other diseases, inhibiting the occurrence of ferroptosis can effectively alleviate the disease, offering a novel concept for future drug treatment and development. Despite the rapid growth of ferroptosis research, there are some insurmountable and inevitable challenges that remain to be resolved. First, many puzzles lie in ferroptosis-associated complicated mechanisms and signaling pathways that need to be further explored, although they indeed provide some constructive and meaningful answers for a multitude of questions regarding the survival and death of cells. It should be noted that ferroptosis also leads to unexpected toxicity and injury, raising awareness that such ferroptosis-modulating therapies need careful consideration and conduction. Additionally, robust and unique biomarkers of ferroptosis, analogous to active caspase-3 for apoptosis, have not yet been discovered and identified until now to guide the appropriate therapy for human pathologies. Instead, evaluating and validating a whole cascade of biochemical and genetic/proteic changes to facilitate the detection and tracking ferroptosis will be necessary to definitively distinguish between distinct forms of RCD.

Although some progress has been made, inconsistent findings are frequently observed in cells, organoids, animals, and even patients (Conrad and Pratt, 2019). As mentioned above, most clinical trials on administering iron chelators and antioxidants have showed only moderate treatment effect, compared to corresponding preclinical studies (Grolez et al., 2015). DFP seems to somewhat slow disease progression and improve quality of life, but they have not reach significance (Martin-Bastida et al., 2017; Klopstock et al., 2019). Unlike mice with a deadly global GPX4-deficiency, SLC7A11-deficient mice have a normal lifespan and do not display any major phenotype develop (Sato et al., 2005; Conrad and Pratt, 2019). However, almost all cells isolated from these mice die rapidly on account of cysteine starvation, GSH depletion, lipid peroxidation and ferroptotic effects that be induced by erastin or other system x_c_^-^-inhibitors. Actully, cysteine is mainly present in the culture medium in its oxidized dimeric form, cystine which can be transported into cells only by system x_c_^-^. Whereas, cysteine is still available in its reduced monomeric form in plasma and other extracellular body fluids in a whole organism, which can be uptaken by cells via neutral amino acid transporters instead of system x_c_^-^ (Conrad and Pratt, 2019). Collectively, the relevance of results from various models require for careful evaluation, owing to the physiological, histological and enzymological differences on specific conditions.

As listed and discussed, some synthetic drugs and natural products have suggested to overcome and treat diseases by regulating ferroptosis or mixed death mainly in preclinical models. However, a general lack of animal experiments and clinical trials limit their further development as future targeted drugs with ferroptotic properties. Particularly, we have shed light on the potential of natural compounds with multiple targets, multiple mechanisms and relatively safe profiles as ferroptosis inducers or inhibitors for (adjuvant) therapy of diseases from the perspective of integration. Although there is growing evidence that natural products are ideal templates for both single ingredient and multiple-component modern drugs, they are faced with many dilemmas and challenges, including productivity problems, poor pharmacokinetics, and unclear pharmacology. Further studies should be conducted to elaborate the pharmacokinetic properties both *in vitro* and *in vivo* to optimize the best functional ingredients and dosage for clinical therapy.
