# Supplementary material for: Emerging Mechanisms and Disease Implications of Ferroptosis: Potential Applications of Natural Products
Source: Front Cell Dev Biol. 2022 Jan 18;9:774957. doi: 10.3389/fcell.2021.774957 (PMC8804219; doi:10.3389/fcell.2021.774957)
Supplement: Supplementary file 1 [file Table1.docx]

**Table S1. Experimental reagents that induce ferroptosis**

| Functional Target | Compounds | Test models | Mechanisms/Effects | Refs |
| --- | --- | --- | --- | --- |
| System x_c_^-^ | Erastin and its analogues:  Piperazine erastin (PE);  Imidazole ketone erastin (IKE) | B16, BT474, PC9, BJeLR cells;  HT-1080 cells and xenograft mice;  Several HCC, BCL, DLBCL cells and xenograft mic | Inhibiting cystine import;  GSH depletion;  PTGS2 upregulation and lipid peroxidation | ^[^[^1-5^](#_ENREF_1)^]^ |
|  | IMCA | HCT-116 cells;  DLD-1 cells and xenograft mice | Decreasing SLC7A11 via AMPK/mTOR/  p70S6k pathway | ^[^[^6^](#_ENREF_6)^]^ |
| GPX4 | RSL3;  (1S,3R) -RSL3 | B16, ID8, COHBR1, BT474, PC9 cells;  HT-1080, BJeLR cells and xenograft mice | Inhibiting GPX4 activity;  ROS accumulation;  Lipid peroxidation;  GSH depletion | ^[^[^1-3^](#_ENREF_1)^,^ [^7^](#_ENREF_7)^,^ [^8^](#_ENREF_8)^]^ |
|  | DPI7/10/12/13/17/  18/19 | HT-1080, BT474, BJeLR,  B16, PC9 cells | Inhibiting GPX4 activity;  Lipid peroxidation | ^[^[^1-3^](#_ENREF_1)^]^ |
| GSH | DPI2;  Buthionine sulfoximine  (BSO) | B16 cells;  HT-1080, BJeLR cells and xenograft mice; | GSH depletion;  ROS accumulation;  Lipid peroxidation | ^[^[^2^](#_ENREF_2)^,^ [^7^](#_ENREF_7)^]^ |
|  | Cyst(e)inase | HT1080, LNCaP cells;  B16, ID8, DU145, PC3, MDA-MB-361, 22Rv1 cells and xenograft mice;  HMVP2 cells and allograft mice;  Cynomolgus monkeys  Primary leukemia cells isolated from CLL leukemia patients;  p53^−/−^ CLL mice; | Cysteine and GSH depletion;  ROS accumulation | ^[^[^7^](#_ENREF_7)^,^ [^9^](#_ENREF_9)^]^ |
|  | AMSNs | L02 cells;  Huh7, MDA-MB 231cells and xenograft mice | Increasing ROS level; ROS accumulation;  Lipid peroxidation | ^[^[^10^](#_ENREF_10)^]^ |
|  | Ce6@MOF | 4T1 cells and xenograft mice | GSH depletion;  GPX4 inactivation | ^[^[^11^](#_ENREF_11)^]^ |
|  | LDL-DHA | PLC/PRF/5, H4IIE cells  HepG2 cells and xenograft mice | Lipid peroxidation; Reducing GPX4 expression | ^[^[^12^](#_ENREF_12)^]^ |
| Iron | Hemoglobin;  FeCl_2_ | OHSCs;  Human iPSC-derived neurons;  ICH model mice | Inducing lethal ROS;  Lipid peroxidation | ^[^[^13^](#_ENREF_13)^]^ |
|  | Hemin | THP-1, IMR-32 cells | ROS generation;  Increasing Fe (II), HO-1 and lipid peroxidation;  Reducing GPX4 activity | ^[^[^14^](#_ENREF_14)^,^ [^15^](#_ENREF_15)^]^ |
|  | (NH_4_)_2_Fe(SO_4_)_2_ | IMR-32 cells | Increasing iron level and lipid peroxidation | ^[^[^15^](#_ENREF_15)^]^ |
|  | Ferric 8-hydroxyquinoline complex (Fe-8HQ) | AML12, HT-1080, HeLa cells | Iron overload and oxidative damage;  Increasing ALOXs and ROS production | ^[^[^16^](#_ENREF_16)^]^ |
|  | Nonthermal plasma (NTP) | IMR 90SV, Rat-1, SM2, EM2 cells | Ferritin destruction;  Lipid ROS production;  Increasing endocytosis, lysosome biogenesis and autophagy | ^[^[^17^](#_ENREF_17)^]^ |
|  | FeGd-HN@Pt@LF/  RGD2 | MCF-7 cells;  U-87 MG cells and xenograft mice | Increasing ROS level | ^[^[^18^](#_ENREF_18)^]^ |
|  | FePt-PTTA-Eu3+-FA (FPEF) | MCF-7, Hela, HepG2 cells;  4T1 cells and xenograft mice | Lipid peroxidation;  Increasing ROS and decreasing GSH | ^[^[^19^](#_ENREF_19)^]^ |
| GPX4;  Iron | FINO_2_ | HT-1080, CAKI-1 cells;  BJ-hTERT cells and its tumorigenic counterpart BJ-eLR cells | GPX4 inactivation;  Oxidizing iron directly;  Lipid peroxidation | ^[^[^20^](#_ENREF_20)^]^ |
| GPX4;  SQS;  CoQ10 | FIN56 | Four engineered BJ cell lines (BJeLR, DRD, BJeHLT, BJeH);  HT-1080 cells | Binding to and activating SQS;  Inhibiting CoQ10 and GPX4 | ^[^[^21^](#_ENREF_21)^]^ |
| Iron;  System x_c_^-^ | MON-p53 | SCC-7, COS7 cells;  HT-1080, 4T1 cells and xenograft mice | Increasing ROS level;  Lipid peroxidation;  GSH depletion;  SLC7A11 and GPX4 downregulation | ^[^[^22^](#_ENREF_22)^]^ |
| Mitochondrial complex I (CI) | BAY 87-2243 | SK-MEL-5, SK-MEL-2, IPC-298, CHL-1, Colo-792 cells;  A-375, G-361, SK-MEL-28, LOX-IMVI cells and xenograft mice;  Patient-derived melanoma mice (MEXF 276 and MEXF 1732) | Δψ Depolarizition;  Increasing cellular ROS;  Lipid peroxidation; GSH depletion | ^[^[^23^](#_ENREF_23)^,^ [^24^](#_ENREF_24)^]^ |
| IκBα | BAY 11-7085 | MDA-MB-231, MDA-MB-468, MCF-7, SKBR3, A549, SKOV3, HuH-7 cells | ROS accumulation;  GSH depletion;  Lipid peroxidation | ^[^[^25^](#_ENREF_25)^]^ |
| FSP1;  CoQ10 | iFSP1 | WT/GPX4-knockout Pfa1 and HT1080 cells (with FSP1 overexpression or not);  A panel of FSP1-knockout human cancer cell lines;  A panel of FSP1 overexpression mouse and human cancer cell lines | Inducing ferroptosis in GPX4-knockout cells and sensitizing to RSL3-induced ferroptosis | ^[^[^26^](#_ENREF_26)^]^ |
| FBXL2;  GPX4 | ALZ003 | A172 cells;  U87MG cells and xenograft mice;  Primary glioblastoma Pt#3 cells;  Mouse primary astrocytes | Blocking GPX4 expression;  Increasing ROS and lipid peroxidation;  Inducing FBXL2-mediated ubiquitination | ^[^[^27^](#_ENREF_27)^]^ |
| Unknown | CIL41/56/69/  70/75/ 79 | Four engineered BJ cell lines (BJeLR, DRD, BJeHLT, BJeH);  HT-1080 cells | Lipid peroxidation | ^[^[^21^](#_ENREF_21)^]^ |
|  | Zinc | A549 cells | Lipid peroxidation;  GSH depletion | ^[^[^28^](#_ENREF_28)^]^ |

HCC, hepatocellular carcinoma; BCL, B cell lymphoma; DLBCL, diffuse large B cell lymphoma; GSH, glutathione; PTGS2, prostaglandin-endoperoxide synthase 2; IMCA, 2-imino-6-methoxy-2H-chromene-3-carbothioamide; SLC7A11, solute carrier family 7 member 11; RSL3, RAS selective lethal 3; GPX4, peroxidase glutathione peroxidase 4; ROS, reactive oxygen species; CLL, chronic lymphocytic; AMSNs, arginine-rich manganese silicate nanobubbles; Ce6@MOF, Ce6-loaded metal organic framework (MOF) nanocarrier; LDL-DHA, low-density lipoprotein nanoparticles reconstituted with the natural omega-3 fatty acid, docosahexaenoic acid; OHSCs, organotypic hippocampal slice cultures; iPSC, induced pluripotent stem cell; ICH, intracerebral hemorrhage; HO-1, heme oxygenase 1; ALOX, lipoxygenase; SQS, squalene synthase; CoQ10, coenzyme Q10; MON-p53, MON encapsulated with p53 plasmid; FSP, ferroptosis suppressor protein; FBXL2, F-box and leucine-rich repeat protein 2; CIL, caspase-3/7-independent lethal.

**Reference**

1 Dixon SJ, Lemberg KM, Lamprecht MR, Skouta R, Zaitsev EM, Gleason CE*, et al*. Ferroptosis: an iron-dependent form of nonapoptotic cell death. Cell 2012; 149: 1060-72.

2 Yang WS, SriRamaratnam R, Welsch ME, Shimada K, Skouta R, Viswanathan VS*, et al*. Regulation of ferroptotic cancer cell death by GPX4. Cell 2014; 156: 317-31.

3 Hangauer MJ, Viswanathan VS, Ryan MJ, Bole D, Eaton JK, Matov A*, et al*. Drug-tolerant persister cancer cells are vulnerable to GPX4 inhibition. Nature 2017; 551: 247-50.

4 Zhang Y, Tan H, Daniels JD, Zandkarimi F, Liu H, Brown LM*, et al*. Imidazole Ketone Erastin Induces Ferroptosis and Slows Tumor Growth in a Mouse Lymphoma Model. Cell Chem Biol 2019; 26: 623-33.e9.

5 Feng H, Schorpp K, Jin J, Yozwiak CE, Hoffstrom BG, Decker AM*, et al*. Transferrin Receptor Is a Specific Ferroptosis Marker. Cell Rep 2020; 30: 3411-23.e7.

6 Zhang L, Liu W, Liu F, Wang Q, Song M, Yu Q*, et al*. IMCA Induces Ferroptosis Mediated by SLC7A11 through the AMPK/mTOR Pathway in Colorectal Cancer. Oxidative medicine and cellular longevity 2020; 2020: 1675613.

7 Wang W, Green M, Choi JE, Gijon M, Kennedy PD, Johnson JK*, et al*. CD8(+) T cells regulate tumour ferroptosis during cancer immunotherapy. Nature 2019; 569: 270-4.

8 Viswanathan VS, Ryan MJ, Dhruv HD, Gill S, Eichhoff OM, Seashore-Ludlow B*, et al*. Dependency of a therapy-resistant state of cancer cells on a lipid peroxidase pathway. Nature 2017; 547: 453-7.

9 Cramer SL, Saha A, Liu J, Tadi S, Tiziani S, Yan W*, et al*. Systemic depletion of L-cyst(e)ine with cyst(e)inase increases reactive oxygen species and suppresses tumor growth. Nature medicine 2017; 23: 120-7.

10 Wang S, Li F, Qiao R, Hu X, Liao H, Chen L*, et al*. Arginine-Rich Manganese Silicate Nanobubbles as a Ferroptosis-Inducing Agent for Tumor-Targeted Theranostics. ACS nano 2018; 12: 12380-92.

11 Meng X, Deng J, Liu F, Guo T, Liu M, Dai P*, et al*. Triggered All-Active Metal Organic Framework: Ferroptosis Machinery Contributes to the Apoptotic Photodynamic Antitumor Therapy. Nano letters 2019; 19: 7866-76.

12 Ou W, Mulik RS, Anwar A, McDonald JG, He X, Corbin IR. Low-density lipoprotein docosahexaenoic acid nanoparticles induce ferroptotic cell death in hepatocellular carcinoma. Free Radic Biol Med 2017; 112: 597-607.

13 Li Q, Han X, Lan X, Gao Y, Wan J, Durham F*, et al*. Inhibition of neuronal ferroptosis protects hemorrhagic brain. JCI insight 2017; 2: e90777.

14 Imoto S, Kono M, Suzuki T, Shibuya Y, Sawamura T, Mizokoshi Y*, et al*. Haemin-induced cell death in human monocytic cells is consistent with ferroptosis. Transfus Apher Sci 2018; 57: 524-31.

15 Hassannia B, Wiernicki B, Ingold I, Qu F, Van Herck S, Tyurina YY*, et al*. Nano-targeted induction of dual ferroptotic mechanisms eradicates high-risk neuroblastoma. The Journal of clinical investigation 2018; 128: 3341-55.

16 Fang S, Yu X, Ding H, Han J, Feng J. Effects of intracellular iron overload on cell death and identification of potent cell death inhibitors. Biochem Biophys Res Commun 2018; 503: 297-303.

17 Shi L, Ito F, Wang Y, Okazaki Y, Tanaka H, Mizuno M*, et al*. Non-thermal plasma induces a stress response in mesothelioma cells resulting in increased endocytosis, lysosome biogenesis and autophagy. Free Radic Biol Med 2017; 108: 904-17.

18 Shen Z, Liu T, Li Y, Lau J, Yang Z, Fan W*, et al*. Fenton-Reaction-Acceleratable Magnetic Nanoparticles for Ferroptosis Therapy of Orthotopic Brain Tumors. ACS nano 2018; 12: 11355-65.

19 Yue L, Dai Z, Chen X, Liu C, Hu Z, Song B*, et al*. Development of a novel FePt-based multifunctional ferroptosis agent for high-efficiency anticancer therapy. Nanoscale 2018; 10: 17858-64.

20 Gaschler MM, Andia AA, Liu H, Csuka JM, Hurlocker B, Vaiana CA*, et al*. FINO(2) initiates ferroptosis through GPX4 inactivation and iron oxidation. Nat Chem Biol 2018; 14: 507-15.

21 Shimada K, Skouta R, Kaplan A, Yang WS, Hayano M, Dixon SJ*, et al*. Global survey of cell death mechanisms reveals metabolic regulation of ferroptosis. Nature chemical biology 2016; 12: 497-503.

22 Zheng DW, Lei Q, Zhu JY, Fan JX, Li CX, Li C*, et al*. Switching Apoptosis to Ferroptosis: Metal-Organic Network for High-Efficiency Anticancer Therapy. Nano letters 2017; 17: 284-91.

23 Basit F, van Oppen LM, Schockel L, Bossenbroek HM, van Emst-de Vries SE, Hermeling JC*, et al*. Mitochondrial complex I inhibition triggers a mitophagy-dependent ROS increase leading to necroptosis and ferroptosis in melanoma cells. Cell death & disease 2017; 8: e2716.

24 Schockel L, Glasauer A, Basit F, Bitschar K, Truong H, Erdmann G*, et al*. Targeting mitochondrial complex I using BAY 87-2243 reduces melanoma tumor growth. Cancer & metabolism 2015; 3: 11.

25 Chang LC, Chiang SK, Chen SE, Yu YL, Chou RH, Chang WC. Heme oxygenase-1 mediates BAY 11-7085 induced ferroptosis. Cancer Lett 2018; 416: 124-37.

26 Doll S, Freitas FP, Shah R, Aldrovandi M, da Silva MC, Ingold I*, et al*. FSP1 is a glutathione-independent ferroptosis suppressor. Nature 2019; 575: 693-8.

27 Chen TC, Chuang JY, Ko CY, Kao TJ, Yang PY, Yu CH*, et al*. AR ubiquitination induced by the curcumin analog suppresses growth of temozolomide-resistant glioblastoma through disrupting GPX4-Mediated redox homeostasis. Redox Biol 2020; 30: 101413.

28 Palmer LD, Jordan AT, Maloney KN, Farrow MA, Gutierrez DB, Gant-Branum R*, et al*. Zinc intoxication induces ferroptosis in A549 human lung cells. Metallomics : integrated biometal science 2019; 11: 982-93.
