# Supplementary material for: Emerging Mechanisms and Disease Implications of Ferroptosis: Potential Applications of Natural Products
Source: Front Cell Dev Biol. 2022 Jan 18;9:774957. doi: 10.3389/fcell.2021.774957 (PMC8804219; doi:10.3389/fcell.2021.774957)
Supplement: Supplementary file 2 [file Table2.docx]

**Table S2.** **Experimental reagents that inhibit ferroptosis**

| Functional Target | Compounds | Test models | Mechanisms/Effects | Refs |
| --- | --- | --- | --- | --- |
| (ROS from) lipid peroxidation | D-PUFAs | BJeLR, HT-108, G-401 cells | Blocking lipid peroxidation | ^[^[^1^](#_ENREF_1)^]^ |
|  | XJB-5-131;  JP4-039 | HT-1080, BJeLR, PANC-1 cells | Preventing mitochondrial lipid oxidation | ^[^[^2^](#_ENREF_2)^]^ |
|  | Ferrostatin-1  (Fer-1) | HT-1080, BT474, HT-22, HEK-  293, A375 cells;  OHSCs, MEFs, Oligodendrocytes;  Proximal renal tubular cells;  Human iPSCs-derived neurons; LOXIMVI cells and mice  ICH model mice | Preventing ROS accumulation and lipid peroxidation;  Reversing ferroptosis caused by GPX4 inhibition;  Downregulating COX2 | ^[^[^3-8^](#_ENREF_3)^]^ |
|  | SSRS11-92 | HT-1080 cells;  HD brain-slice;  Oligodendrocytes;  Proximal renal tubular cells | Inhibiting lipid peroxidation | ^[^[^8^](#_ENREF_8)^]^ |
|  | SRS 16-86 | HT-29, NIH 3T3, HT1080 cells;  I/R model mice | Increasing healthy spinous neurons;  Inhibiting oxidative lipid damage and ferroptosis;  Strong protection against I/R | ^[^[^9^](#_ENREF_9)^]^ |
|  | Liproxstatin-1  (Lip-1) | HK-2, HT-22, HEK-293 cells;  MEFs, HRPTEpiCs;  HT-1080, B16 and ID8 xenograft mice;  ALOX15/GPX4 DKO mice;  I/R-induced hepatic damage mice;  Chronic morphine tolerance model mice | Reversing anit-tumor efficacy of ferroptsis inducers;  Reducing iron overload and lipid peroxidation;  Reducing MDA and ROS levels;  Increasing GPX4 level | ^[^[^4^](#_ENREF_4)^,^ [^10-12^](#_ENREF_10)^]^ |
|  | A series of synthesized hydroxylated chalcones | SH-SY5Y cells | Inhibiting amyloid-β peptide aggregation;  Inhibiting lipid peroxidation | ^[^[^13^](#_ENREF_13)^]^ |
| Antioxidants | THNs | HEK-293 cells;  MEFs | Inhibiting lipid peroxidation | ^[^[^4^](#_ENREF_4)^]^ |
|  | Butylated hydroxyltoluene  (BHT) | HT1080 cells | Preventing ROS production and cell death | ^[^[^5^](#_ENREF_5)^]^ |
|  | Trolox | HT1080, Calu-1, BJeLR cells;  MEFs | Eliminating ROS | ^[^[^5^](#_ENREF_5)^]^ |
|  | GIF-0726-r | HT22 cells | Activating ARE;  Inhibiting ROS accumulation and Ca^2+^ influx | ^[^[^14^](#_ENREF_14)^]^ |
|  | Diarylamine;  Phenoxazine;  Phenothiazine | HepG2 cells, MEFs | Inhibiting lipid peroxidation | ^[^[^15^](#_ENREF_15)^]^ |
|  | PMC | HEK-293 cells | Inhibiting lipid peroxidation | ^[^[^16^](#_ENREF_16)^]^ |
|  | TEMPO | MEFs | Inhibiting lipid peroxidation | ^[^[^17^](#_ENREF_17)^]^ |
| ALOX | PD146176 | HT-1080, BT474, A375 cells | Preventing RSL3-induced ferroptosis;  Reversing ferroptosis caused by GPX4 inhibition | ^[^[^1^](#_ENREF_1)^,^ [^3^](#_ENREF_3)^,^ [^18^](#_ENREF_18)^]^ |
|  | CDC;  AA-861 | HT1080 cells | Rescuing cells from erastin-induced ferroptosis | ^[^[^1^](#_ENREF_1)^]^ |
|  | BW A4C | MEFs | Inhibiting lipid peroxidation | ^[^[^19^](#_ENREF_19)^]^ |
| Iron | 2,2’-dipyridyl | HT1080 cells | Substantial protection from death | ^[^[^5^](#_ENREF_5)^]^ |
| System x_c_^-^ | β-mercaptoethanol  (β-ME) | HT1080 cells | Increasing cysteine for GSH synthesis | ^[^[^5^](#_ENREF_5)^]^ |
| NOX | Diphenylene  Iodonium (DPI);  GKT137831 | HT1080, Calu-1 cells | Inhibiting NOX;  Suppressing erastin-induced ferroptosis | ^[^[^5^](#_ENREF_5)^]^ |
| Pentose phosphate pathway (PPP) | 6-aminonicotinamde  (6-AN) | HT1080, Calu-1, BJeLR cells | Inhibiting NADPH-generated PPP;  Preventing ROS production and cell death | ^[^[^5^](#_ENREF_5)^]^ |
| Lysosomes | Ammonium chloride;  Baf A_1_;  PepA-Me | HT1080, Calu-1 cells | Inhibiting iron accumulation and ROS generation;  Protecting cells from erastin- or RSL3-induced feroptosis | ^[^[^20^](#_ENREF_20)^]^ |
| GPX | Two new probucol analogues | HT22 cells;  Primary cortical  neurons | Decreasing mitochondrial superoxide and hyperpolarization;  Increasing GPX activity | ^[^[^21^](#_ENREF_21)^]^ |
| GLS2 | Compound 968 | MEFs;  I/R model mice | An inhibitor of GLS;  Inhibiting LDH release | ^[^[^22^](#_ENREF_22)^]^ |
| Transaminase | Aminooxyacetic acid (AOA) | HT-1080, BJeLR, HT-22 cells | Blocking the metabolism of glutamine to α-KG for fatty acid synthesis  Rescuing cells from ferroptosis | ^[^[^5^](#_ENREF_5)^]^ |
| Lipid transporter SCP2 | SCPI-2 | BT474, A375 cells | Inhibiting lipid hydroperoxides;  Reversing ferroptosis caused by GPX4 inhibition | ^[^[^3^](#_ENREF_3)^]^ |
| PDK1 | GSK2334470 | HT-1080 cells | Reducing iron overload;  Suppressing RSL3-  induced lipid ROS | ^[^[^18^](#_ENREF_18)^]^ |
| PKC | BisIII | LUHMES cells  Organotypic slices cultures | Counteracting erastin-induced ferroptosis | ^[^[^23^](#_ENREF_23)^]^ |
| JNK | SP600125 | HD brain-slice;  HL-60 cells | Inhibiting oxidative lipid damage | ^[^[^8^](#_ENREF_8)^,^ [^24^](#_ENREF_24)^]^ |
| p38 | SB202190 | HL-60 cells | Reducing erastin-induced ferroptosis | ^[^[^24^](#_ENREF_24)^]^ |
| STAT3 | S3I-201 | PANC-1, CFPAC1 cells | Inhibiting STAT3;  Blocking erastin-induced ferroptosis | ^[^[^25^](#_ENREF_25)^]^ |
| Selenoproteins | Supplementation with insulin, transferrin, and selenium (ITS) | H295R cells | Increasing GPX4 level and selenoproteins | ^[^[^26^](#_ENREF_26)^]^ |
|  | Selenium (Se);  A selenocysteine-containing peptide, Tat SelPep | HT22, HT1080 cells;  Immature and mature primary cortical neuronal cultures;  Collagenase-induced ICH and acute ischemic stroke mice | Increasing selenoproteins;  Inhibiting GPX4-dependent ferroptosis | ^[^[^27^](#_ENREF_27)^]^ |

D-PUFA, deuterated polyunsaturated fatty acids; OHSCs, organotypic hippocampal slice cultures; MEF, mouse embryonic fibroblast; iPSC, induced pluripotent stem cell; ICH, intracerebral hemorrhage; ROS, reactive oxygen species; GPX4, glutathione peroxidase 4; COX2, cyclooxygenase 2; HD, Huntington’s disease; I/R, ischemia-reperfusion; HRPTEpiC, human renal proximal tubule epithelial cell; ALOX, lipoxygenase; DKO, double-knockout; MDA, malondialdehyde; THN, tetrahydronapthyridinol; ARE, antioxidant response element; PMC, 2,2,7,8-pentamethyl-6-chromanol; TEMPO, 2,2,6,6-tetramethylpiperidin-N-oxyl; RSL3, RAS selective lethal 3; CDC, cinnamyl-3,4-dihydroxya-cyanocinnamate; GSH, glutathione; NOX, nicotinamide adenine dinucleotide phosphate (NADPH) oxidase; PPP, pentose phosphate pathway; GLS, glutaminases; LDH, lactate dehydrogenase; α-KG, alpha-ketoglutarate; PDK1, 3-phosphoinositidedependent kinase 1; PKC, protein kinase C; STAT3, signal transducer and activator of transcription 3.

**Reference**

1 Yang WS, Kim KJ, Gaschler MM, Patel M, Shchepinov MS, Stockwell BR. Peroxidation of polyunsaturated fatty acids by lipoxygenases drives ferroptosis. Proc Natl Acad Sci U S A 2016; 113: E4966-75.

2 Krainz T, Gaschler MM, Lim C, Sacher JR, Stockwell BR, Wipf P. A Mitochondrial-Targeted Nitroxide Is a Potent Inhibitor of Ferroptosis. ACS Cent Sci 2016; 2: 653-9.

3 Hangauer MJ, Viswanathan VS, Ryan MJ, Bole D, Eaton JK, Matov A*, et al*. Drug-tolerant persister cancer cells are vulnerable to GPX4 inhibition. Nature 2017; 551: 247-50.

4 Zilka O, Shah R, Li B, Friedmann Angeli JP, Griesser M, Conrad M*, et al*. On the Mechanism of Cytoprotection by Ferrostatin-1 and Liproxstatin-1 and the Role of Lipid Peroxidation in Ferroptotic Cell Death. ACS Cent Sci 2017; 3: 232-43.

5 Dixon SJ, Lemberg KM, Lamprecht MR, Skouta R, Zaitsev EM, Gleason CE*, et al*. Ferroptosis: an iron-dependent form of nonapoptotic cell death. Cell 2012; 149: 1060-72.

6 Li Q, Han X, Lan X, Gao Y, Wan J, Durham F*, et al*. Inhibition of neuronal ferroptosis protects hemorrhagic brain. JCI insight 2017; 2: e90777.

7 Viswanathan VS, Ryan MJ, Dhruv HD, Gill S, Eichhoff OM, Seashore-Ludlow B*, et al*. Dependency of a therapy-resistant state of cancer cells on a lipid peroxidase pathway. Nature 2017; 547: 453-7.

8 Skouta R, Dixon SJ, Wang J, Dunn DE, Orman M, Shimada K*, et al*. Ferrostatins inhibit oxidative lipid damage and cell death in diverse disease models. Journal of the American Chemical Society 2014; 136: 4551-6.

9 Linkermann A, Skouta R, Himmerkus N, Mulay SR, Dewitz C, De Zen F*, et al*. Synchronized renal tubular cell death involves ferroptosis. Proc Natl Acad Sci U S A 2014; 111: 16836-41.

10 Wang W, Green M, Choi JE, Gijon M, Kennedy PD, Johnson JK*, et al*. CD8(+) T cells regulate tumour ferroptosis during cancer immunotherapy. Nature 2019; 569: 270-4.

11 Chen X, Zhang B, Liu T, Feng M, Zhang Y, Zhang C*, et al*. Liproxstatin-1 Attenuates Morphine Tolerance through Inhibiting Spinal Ferroptosis-like Cell Death. ACS Chem Neurosci 2019; 10: 4824-33.

12 Friedmann Angeli JP, Schneider M, Proneth B, Tyurina YY, Tyurin VA, Hammond VJ*, et al*. Inactivation of the ferroptosis regulator Gpx4 triggers acute renal failure in mice. Nature cell biology 2014; 16: 1180-91.

13 Cong L, Dong X, Wang Y, Deng Y, Li B, Dai R. On the role of synthesized hydroxylated chalcones as dual functional amyloid-β aggregation and ferroptosis inhibitors for potential treatment of Alzheimer's disease. Eur J Med Chem 2019; 166: 11-21.

14 Hirata Y, Yamada C, Ito Y, Yamamoto S, Nagase H, Oh-Hashi K*, et al*. Novel oxindole derivatives prevent oxidative stress-induced cell death in mouse hippocampal HT22 cells. Neuropharmacology 2018; 135: 242-52.

15 Shah R, Margison K, Pratt DA. The Potency of Diarylamine Radical-Trapping Antioxidants as Inhibitors of Ferroptosis Underscores the Role of Autoxidation in the Mechanism of Cell Death. ACS Chem Biol 2017; 12: 2538-45.

16 Shah R, Shchepinov MS, Pratt DA. Resolving the Role of Lipoxygenases in the Initiation and Execution of Ferroptosis. ACS Cent Sci 2018; 4: 387-96.

17 Griesser M, Shah R, Van Kessel AT, Zilka O, Haidasz EA, Pratt DA. The Catalytic Reaction of Nitroxides with Peroxyl Radicals and Its Relevance to Their Cytoprotective Properties. Journal of the American Chemical Society 2018; 140: 3798-808.

18 Fang S, Yu X, Ding H, Han J, Feng J. Effects of intracellular iron overload on cell death and identification of potent cell death inhibitors. Biochem Biophys Res Commun 2018; 503: 297-303.

19 Angeli JPF, Shah R, Pratt DA, Conrad M. Ferroptosis Inhibition: Mechanisms and Opportunities. Trends in pharmacological sciences 2017; 38: 489-98.

20 Torii S, Shintoku R, Kubota C, Yaegashi M, Torii R, Sasaki M*, et al*. An essential role for functional lysosomes in ferroptosis of cancer cells. The Biochemical journal 2016; 473: 769-77.

21 Bueno DC, Canto RFS, de Souza V, Andreguetti RR, Barbosa FAR, Naime AA*, et al*. New Probucol Analogues Inhibit Ferroptosis, Improve Mitochondrial Parameters, and Induce Glutathione Peroxidase in HT22 Cells. Mol Neurobiol 2020.

22 Gao M, Monian P, Quadri N, Ramasamy R, Jiang X. Glutaminolysis and Transferrin Regulate Ferroptosis. Molecular cell 2015; 59: 298-308.

23 Do Van B, Gouel F, Jonneaux A, Timmerman K, Gele P, Petrault M*, et al*. Ferroptosis, a newly characterized form of cell death in Parkinson's disease that is regulated by PKC. Neurobiology of disease 2016; 94: 169-78.

24 Yu Y, Xie Y, Cao L, Yang L, Yang M, Lotze MT*, et al*. The ferroptosis inducer erastin enhances sensitivity of acute myeloid leukemia cells to chemotherapeutic agents. Mol Cell Oncol 2015; 2: e1054549.

25 Gao H, Bai Y, Jia Y, Zhao Y, Kang R, Tang D*, et al*. Ferroptosis is a lysosomal cell death process. Biochemical and biophysical research communications 2018; 503: 1550-6.

26 Belavgeni A, Bornstein SR, von Massenhausen A, Tonnus W, Stumpf J, Meyer C*, et al*. Exquisite sensitivity of adrenocortical carcinomas to induction of ferroptosis. Proc Natl Acad Sci U S A 2019; 116: 22269-74.

27 Alim I, Caulfield JT, Chen Y, Swarup V, Geschwind DH, Ivanova E*, et al*. Selenium Drives a Transcriptional Adaptive Program to Block Ferroptosis and Treat Stroke. Cell 2019; 177: 1262-79 e25.
